# Design of novel interlocked bi-layer NiTi braided stent with ultra-thin walls for urinary tract obstruction treatment

**DOI:** 10.1093/rb/rbag130

**Published:** 2026-06-15

**Authors:** Wenshuo Zhao, Jianjin Wang, Chenglong Yu, Yuecheng Yu, Xiaoli Liu, Jie Qiao, Sanieng Lei, Dong Cao, Wenping Jian, Langda Xu, Fan Zhao, Jing Lin, Chaojing Li, Fujun Wang, Aijun Guo, Lu Wang

**Affiliations:** Key Laboratory of Textile Science & Technology, Ministry of Education, College of Textiles, Donghua University, Shanghai 201620, China; Key Laboratory of Textile Industry for Biomedical Textile Materials and Technology, Donghua University, Shanghai 201620, China; Shanghai Frontiers Science Center of Advanced Textiles, College of Textiles, Donghua University, Shanghai 201620, China; Honest Medical China Co., Ltd., Guangdong 519000, China; Ushare Medical Inc., Guangdong 519040, China; Key Laboratory of Textile Science & Technology, Ministry of Education, College of Textiles, Donghua University, Shanghai 201620, China; Key Laboratory of Textile Industry for Biomedical Textile Materials and Technology, Donghua University, Shanghai 201620, China; Key Laboratory of Textile Science & Technology, Ministry of Education, College of Textiles, Donghua University, Shanghai 201620, China; Key Laboratory of Textile Industry for Biomedical Textile Materials and Technology, Donghua University, Shanghai 201620, China; Key Laboratory of Textile Science & Technology, Ministry of Education, College of Textiles, Donghua University, Shanghai 201620, China; Honest Medical China Co., Ltd., Guangdong 519000, China; Ushare Medical Inc., Guangdong 519040, China; Key Laboratory of Textile Science & Technology, Ministry of Education, College of Textiles, Donghua University, Shanghai 201620, China; Key Laboratory of Textile Science & Technology, Ministry of Education, College of Textiles, Donghua University, Shanghai 201620, China; Key Laboratory of Textile Science & Technology, Ministry of Education, College of Textiles, Donghua University, Shanghai 201620, China; Shanghai Frontiers Science Center of Advanced Textiles, College of Textiles, Donghua University, Shanghai 201620, China; Key Laboratory of Textile Science & Technology, Ministry of Education, College of Textiles, Donghua University, Shanghai 201620, China; Key Laboratory of Textile Industry for Biomedical Textile Materials and Technology, Donghua University, Shanghai 201620, China; Key Laboratory of Textile Science & Technology, Ministry of Education, College of Textiles, Donghua University, Shanghai 201620, China; Shanghai Frontiers Science Center of Advanced Textiles, College of Textiles, Donghua University, Shanghai 201620, China; Key Laboratory of Textile Science & Technology, Ministry of Education, College of Textiles, Donghua University, Shanghai 201620, China; Shanghai Frontiers Science Center of Advanced Textiles, College of Textiles, Donghua University, Shanghai 201620, China; Ushare Medical Inc., Guangdong 519040, China; Key Laboratory of Textile Science & Technology, Ministry of Education, College of Textiles, Donghua University, Shanghai 201620, China; Key Laboratory of Textile Industry for Biomedical Textile Materials and Technology, Donghua University, Shanghai 201620, China

**Keywords:** ureteral stent, braiding, bi-layer structure, mechanical performances, drainage

## Abstract

Conventional ureteral stents often face a critical trade-off between radial support and lumen patency, compromising drainage performance under compression. To overcome this, we designed a novel interlocked bi-layer nitinol (NiTi) braided stent. A thread-inspired interlocking strategy between the inner and outer layers was achieved by introducing a thick NiTi wire into the inner layer, significantly enhancing interlayer bonding (64.10 ± 5.62 cN). The optimized stent featured an ultra-thin wall structure (0.31 ± 0.03 mm), which enabled a 29.03% larger lumen diameter while providing a 174.38% higher radial strength (2215.44 ± 42.87 cN) compared to commercial polyurethane (PU) ureteral stents. Moreover, it demonstrated superior flexibility, with a bending stiffness of 0.87 ± 0.07 N·mm^2^. Under simulated ureteral obstruction model (1.0 mm constant compression) with low renal pressure, the stent preserved 33.56% of its initial drainage rate, while the PU stent lost virtually all flow. Additionally, the retention force (24.08 ± 0.98 cN), optimized via J-end geometry, was equivalent to clinical standards. This study introduces a structurally reinforced, drainage-efficient ureteral stent that synergistically improves lumen patency, mechanical robustness, and anchoring performance. The proposed interlocked bi-layer braiding concept offers a scalable design strategy for next-generation stents and other minimally invasive tubular implants.

## Graphical abstract

**Graphical Abstract:**
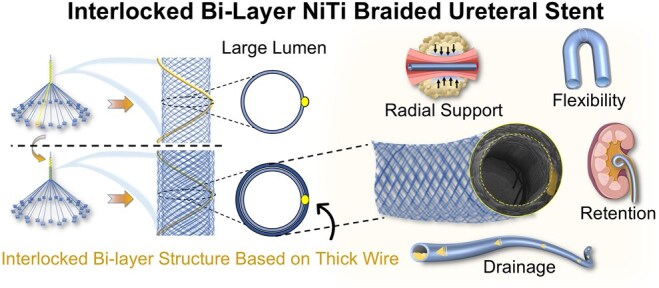

## Introduction

Ureteral stents are essential for treating urinary obstructions, playing a role in supporting the ureter against the compression and draining the urine to ensure normal renal function [1–3]. Ureteral stents are in contact with the surrounding tissue and subjected to various complex clinical stresses, including compression and bending [4–9]. An ideal ureteral stent structure must provide a large lumen diameter with robust radial support to resist collapse under external pressure and maintain lumen patency [10, 11]. To minimize the risk of tissue damage caused by chronic outward forces, the stent should possess excellent flexibility to conform to the tortuous anatomy of the ureter, whilst ensuring that its diameter matches that of the ureteral lumen [12–14].

Currently, the dominant clinical products are double-J (D-J) stents (4–8 Fr) made of polymer materials such as polyurethane (PU) and silicone. Polymeric ureteral stents typically offer excellent flexibility while providing adequate radial support to accommodate most clinical implantation requirements. Although polymeric stents have been widely accepted, they are confronted with a structural contradiction in terms of radial support and lumen patency [15]. Insufficient radial support of the ureteral stent may lead to the loss of lumen patency under compression, which in turn causes secondary obstruction. In order to meet the adequate radial support required for implantation, the polymeric stents’ wall thickness usually needs to be increased [16], which inevitably reduces the inner diameter (ID) of the stent and then compromises the drainage efficiency [17, 18]. To enhance the stability to withstand high-pressure conditions, metallic stent products (18–30 Fr) fabricated from nitinol (NiTi), including Resonance, Urexel and Uventa, have been widely used. Compared with polymeric stents, metallic stents demonstrate enhanced radial support [19], and display higher patency rates than polymeric stents [20, 21]. However, they have the larger caliber and the greater stiffness, which results in a high rate of complications including poor patient tolerance, hematuria and stent migration [22, 23]. Although metallic stent Resonance mainly consists of a coil spring and has the same diameter as the most commonly used size (6 Fr) of polymeric stent, offering strong support and flexibility, its overall drainage performance is inferior to that of polymeric stents [24]. Therefore, there is a compelling need for a novel stent architecture that reconciles these conflicting requirements including low wall thickness and excellent mechanical properties while maintaining the commonly used diameter.

Braiding, a traditional textile technique, is widely used in clinic for manufacturing stents for medical stents [25–27]. By manipulating the material and parameter [28–33], it is possible to create tubular braided fabrics with various morphologies and properties. The selection of metal materials for high-density braiding is a common choice for high radial support [34, 35]. Curing coatings or covering treatments have also been shown to improve radial support to some extent at the expense of stent flexibility [36–38], and destroy the characteristic of the braiding structure. Constructing a bi-layer stent is an effective way to improve the mechanical performance of stents, which allows for a wider variety of wire materials and thicknesses. Bi-layer structure could improve mechanical performance regardless of braiding parameters and other treatments [39]. Nevertheless, there is no significant bonding force between the inner and outer layers of the bi-layer stent, which leads to a potential risk of detrimental deformation during application [40].

In this study, by introducing a thick NiTi wire into the inner layer of the NiTi braided bi-layer stent, a thread-engaged structure between the inner and outer layers was constructed. The interlocked bi-layer stent maintained superior radial support and flexibility to the commercial PU stent, while the larger lumen diameter endowed by the thinner wall had a significant advantage in drainage performance. Furthermore, the retention force of the stent could be adjusted to be equivalent to that of the commercial PU stent, which provided a reference for the design of subsequent stents with J-shaped ends.

## Materials and methods

### Materials and fabrication of the NiTi braided Bi-layer ureteral stent

**Figure 1:**
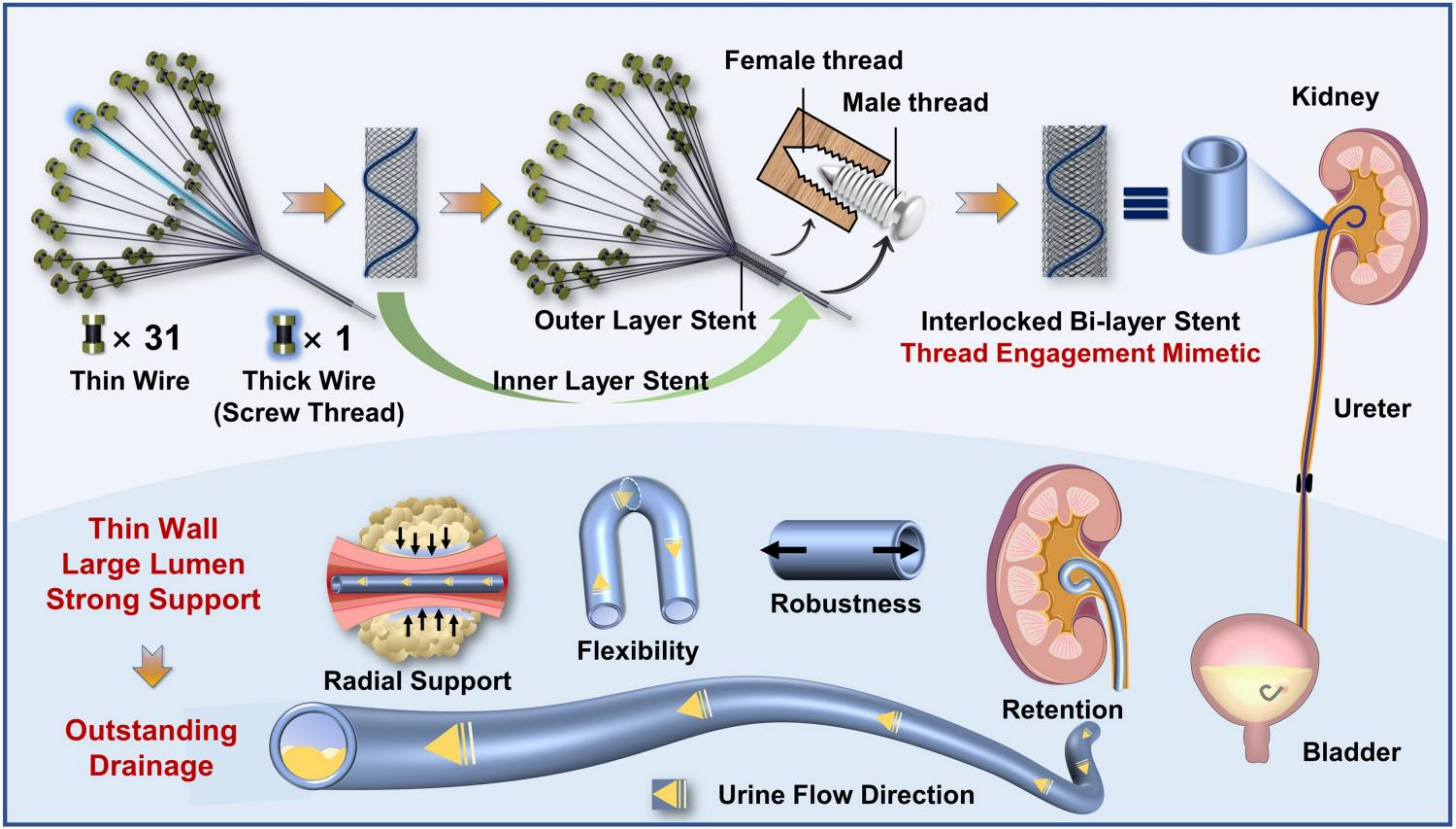
Schematic diagram of the braiding process and design concept of the novel interlocked bi-layer NiTi braided stent.

Superelastic NiTi (d = 0.04 mm, 0.065 mm, 0.08 mm, 0.15 mm) wires were obtained from Ushare Medical Inc. (China). The material composition and superelastic stress–strain curves of NiTi wires were included in Supplementary Table S1 and Supplementary Figure S1. All of the ureteral stents presented in this article were braided by 32 strands of NiTi wires on a 32-ingot horizontal braiding machine (Guan Bo China Machinery). The braiding process was shown in Figure 1.

NiTi wires (diameter, 0.08 mm, 0.065 mm and 0.04 mm) were braided around a stainless steel mandrel with different braiding densities (picks per inch, PPI), to obtain three groups of mono-layer stents (Table 1). Three mono-layer stents were selected as the inner layer stents. Stent M4-4 was denoted as the outer layer stent and braided around the inner layer stent to form the bi-layer stent (Table 2). Based on the bi-layer stent B4, two types of interlocked bi-layer stents were prepared by replacing one thin wire (0.04 mm) in the inner layer stent with an alternative thick wire (0.08 mm or 0.15 mm) for braiding (Table 3). The stent with a stainless steel mandrel underwent preliminary thermal treatment (500°C, 1 min) in muffle furnace (LT40/11/B410, Nabertherm Co., Ltd., Lilienthal, Germany), water quench (20°C) and air-drying at room temperature. After that, a second thermal treatment (500°C, 5 min) followed, using a new mandrel (straight or J-shaped) for final shaping. For the superelasticity test, NiTi wires were also thermal treated (500°C, 5 min).

**Table 1:** Braiding parameters of mono-layer stents.

Sample	Wire diameter (mm)/PPI	Sample	Wire diameter (mm)/PPI	Sample	Wire diameter (mm)/PPI
M8-1	0.08/30	M65-1	0.065/40	M4-1	0.04/70
M8-2	0.08/35	M65-2	0.065/45	M4-2	0.04/90
M8-3	0.08/40	M65-3	0.065/50	M4-3	0.04/100
M8-4	0.08/45	M65-4	0.065/55	M4-4	0.04/110

**Table 2:** Braiding parameters of bi-layer stents.

Sample	Wire diameter (mm)/PPI	
	Inner layer	Outer layer
B8	0.08/40	0.04/110
B65	0.065/50	0.04/110
B4	0.04/110	0.04/110

**Table 3:** Braiding parameters of interlocked bi-layer stents.

Sample	Wire diameter (mm)/PPI	
	Inner layer	Outer layer
B4	0.04/110	0.04/110
B4-8	0.04/110 + 0.08	0.04/110
B4-15	0.04/110 + 0.15	0.04/110

### Characterization of stent structural parameters

The morphology of stents was observed using a scanning electron microscope (SEM, Hitachi TM3000, Japan). Software Image J was used to measure the outer diameter (OD), braiding angle (α), pitch, PPI according to the microscope image and calculate the wall thickness.

#### Bending test

For the qualitative bending test, two short rods with a diameter of 1.6 mm were inserted into both ends of the stent, the distance between ends two inserted of rods was 25 mm. The stent was bent by turning the short rod in the horizontal plane, and the outermost ends of the stent were 15 mm apart, which was controlled by the positioning pins, and the lumen morphology at the bend was observed. The quantitative bending test was finished on a semi-automatic bending tester (KES-FB2S, Kato Tech Co., Ltd., Japan). Test conditions: curvature range 0.05–0.15 mm^−1^, clamping plate spacing 10 mm, bending rate of 0.05 mm^−1^/s, measurement range of 5 N·mm.

#### Axial stretching test

The axial tensile property was tested using a textile multifunctional strength testing machine (YG(B) 026G-500, Wenzhou Darong Textile Instrument Co., Ltd., China). For the test of the wire, referring to standard YS/T 1147-2016, the stretching velocity was 1 mm/min, the spacing was 100 mm and the tensile strain was 6%. For the test of the stent, referring to YY/T 0872-2013, the stretching velocity was 200 mm/min and the spacing was 50 mm. Short metal support bars with a diameter of 1.6 mm were inserted into both ends of the stent during the test.

#### Radial compression test

The compression performance of stents was measured by a compression elasticity tester (LLY-06D, Laizhou Electronic Instrument Co., Ltd., China). Referring to ISO 25539-2-2012, plate compression and partial compression methods were used with consideration of the stent structure. Test conditions: sample length 40 mm, presser foot diameter 50 mm (plate compression) and 3 mm (partial compression), compression displacement 50% ID, displacement velocity 10 mm/min. The compression strength was recorded when the presser foot reached the compression displacement from the top of the stent, and subsequently the presser foot remained stationary for 10 s before unloading.

#### Interlayer bonding test

The bonding force between two layers of the stent was measured by stretching the inner and outer layer respectively with a displacement velocity of 10 mm/min on a computerized tension and pressure testing machine (HY-940FS, Shanghai Hengyu Instrument Co., Ltd., China).

#### Fatigue test

The stent (21 cm) was inserted into a silicone tube (ID = 3 mm, OD = 5 mm) and filled with phosphate buffer saline (PBS) using a syringe; the silicone tube was then sealed to prevent leakage. Fatigue test was conducted on custom-built tensile and torsion fatigue testing device [41], with both ends of the silicone tube clamped and moved up and down by the jaws to perform repetitive bending. The jaw performed a cyclic motion with a vertical travel of 200 mm at a rate of 0.1 cycles per second, thereby altering the bending state of the stent.

#### Drainage test

Referring to previous reports [28, 42], a modified drainage test system was built, which consisted of several parts (Figure 6A). A reservoir bottle and a liquid flow channel with a 18G single nozzle imitate the kidney. Two thin-wall silicone tubes (ID 2.4 mm) simulate the unobstructed part of the ureter. The sample stent (25 cm) connected with the 18G single nozzle was deployed through two silicone tubes, and the proximal and distal sections of the stent overlapped with these two tubes, respectively. To mimic the obstructed part of the stent, sideholes located in the middle section of the stent were sealed with sealing film to prevent water leakage. To simulate the loss of stent lumen, the compression elasticity tester applied in the radial compression test was deployed to provide compression of constant distances (0 mm, 0.8 mm and 1.0 mm) at the middle section of the stent (presser foot diameter 5 mm). The drainage rate was regulated by varying the difference in height (*h*). The amount of the test liquid drainage collected by a graduated cylinder within 10 min was recorded. Simultaneously, the test liquid in the reservoir was continuously replenished for consistent hydraulic pressure difference.

#### Anti-migration test

The retention force was characterized using a polytetrafluoroethylene (PTFE) mold referring to YY/T 0872-2013 on a computerized tension and pressure testing machine (HY-940FS, Shanghai Hengyu Instrument Co., Ltd., China). The J shaped end was inserted in the mold and the other end was fixed (Figure 7A). The J shaped end was pulled out from the mold at a speed of 500 mm/min. The maximum force during pulling was recorded as the retention force. Several parameters were controlled, including stent diameter (4.5 and 6 Fr), end diameter (10, 11, 12, 13 and 14 mm), number of end loops (1/2, 1, 3/2 and 2), and end pitch (2, 5 and 10 mm). The dimension parameter of the mold was shown in Figure 7B.

### Statistical analysis

The data were analyzed by one-way analysis of variance (ANOVA). The values were expressed as mean ± standard deviation (SD), and significant differences were expressed as * (*P* < 0.05), ** (*P* < 0.01), *** (*P* < 0.001).

## Results and discussion

### Design of novel braided ureteral stent

It is challenging in the design of medical stents to simultaneously achieve satisfactory radial support and flexibility, which are two conflicting properties [32]. Braided stents resist external forces by slip and filament rotation at the interwoven points when subjected to external force. The wire diameter and PPI of the mono-layer braided stent were adjusted to balance the above contradictions; however, the mono-layer braided structure could not provide sufficient radial support. Furthermore, the optimal mono-layer structure was selected as the outer layer and braided around the inner layer. The resultant bi-layer structure significantly improved the mechanical properties, especially the partial compression strength, while maintaining excellent flexibility. To prevent interlayer delamination of the bi-layer stent, a single thick NiTi wire was introduced into the inner layer, and an interlocked bi-layer stent was successfully developed (Figure 1). Detailed information of stent diameter, wall thickness, included angle of two wires (2α), pitch and actual PPI were demonstrated in Supplementary Tables S3–S5.

### Fabrication and characterization of mono-layer braided stents

**Figure 2:**
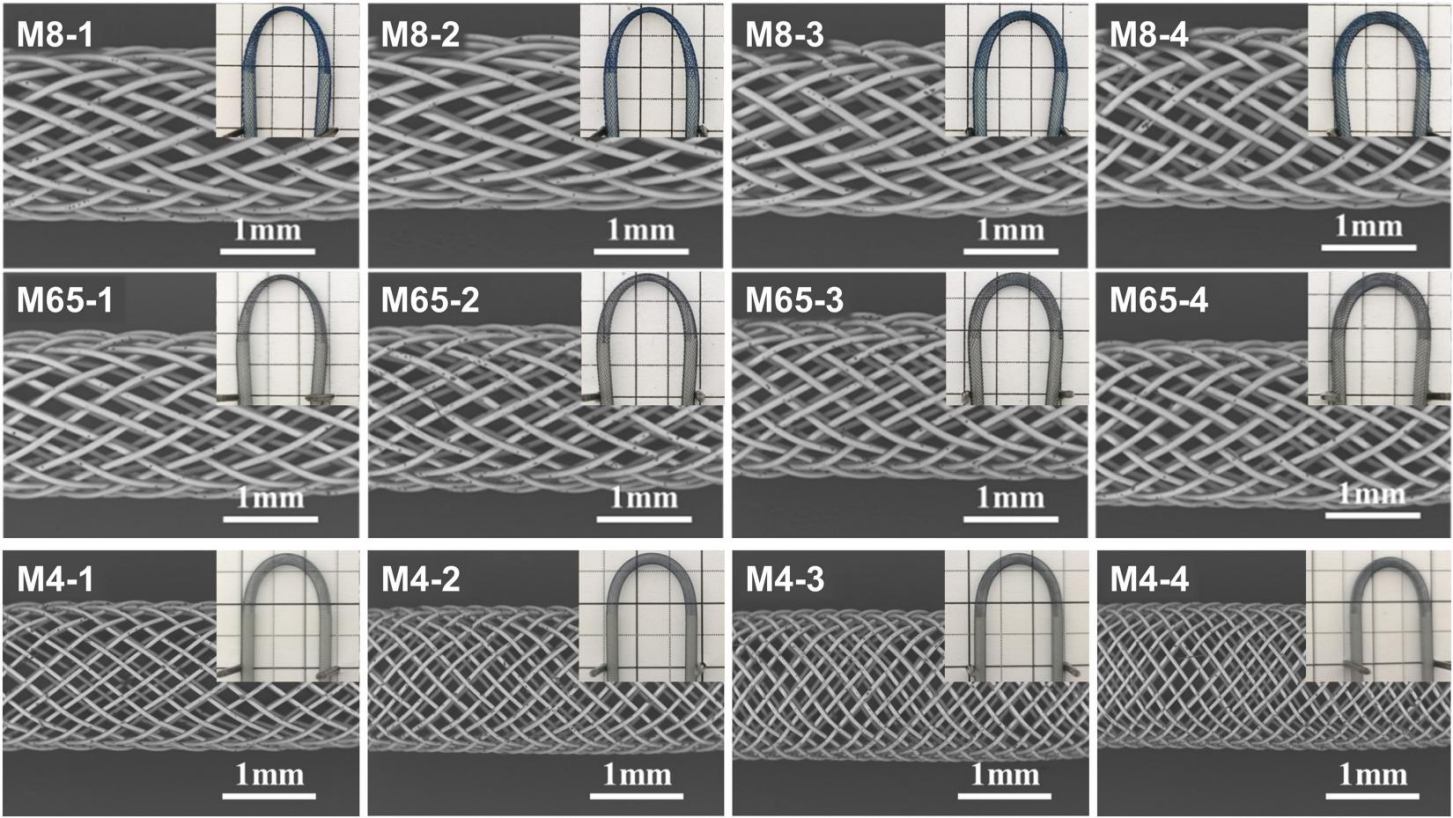
Morphologies under SEM and qualitative bending results of groups M8, M65 and M4. M8-1 to M8-4 were braided by 0.08 mm wires with PPI 30, 35, 40 and 45. M65-1 to M65-4 were braided by 0.065 mm wires with PPI 40, 45, 50 and 55. M4-1 to M4-4 were braided by 0.04 mm wires with PPI 70, 90 100 and 110.

Since NiTi wires with different diameter have different stiffnesses, they are not able to be braided uniformly with the same PPI. Braidable PPI range for wires with different diameters were listed in Supplementary Table S2. All the stents were uniform and stable, and no irregular wire flexure was observed (Figure 2).

**Figure 3:**
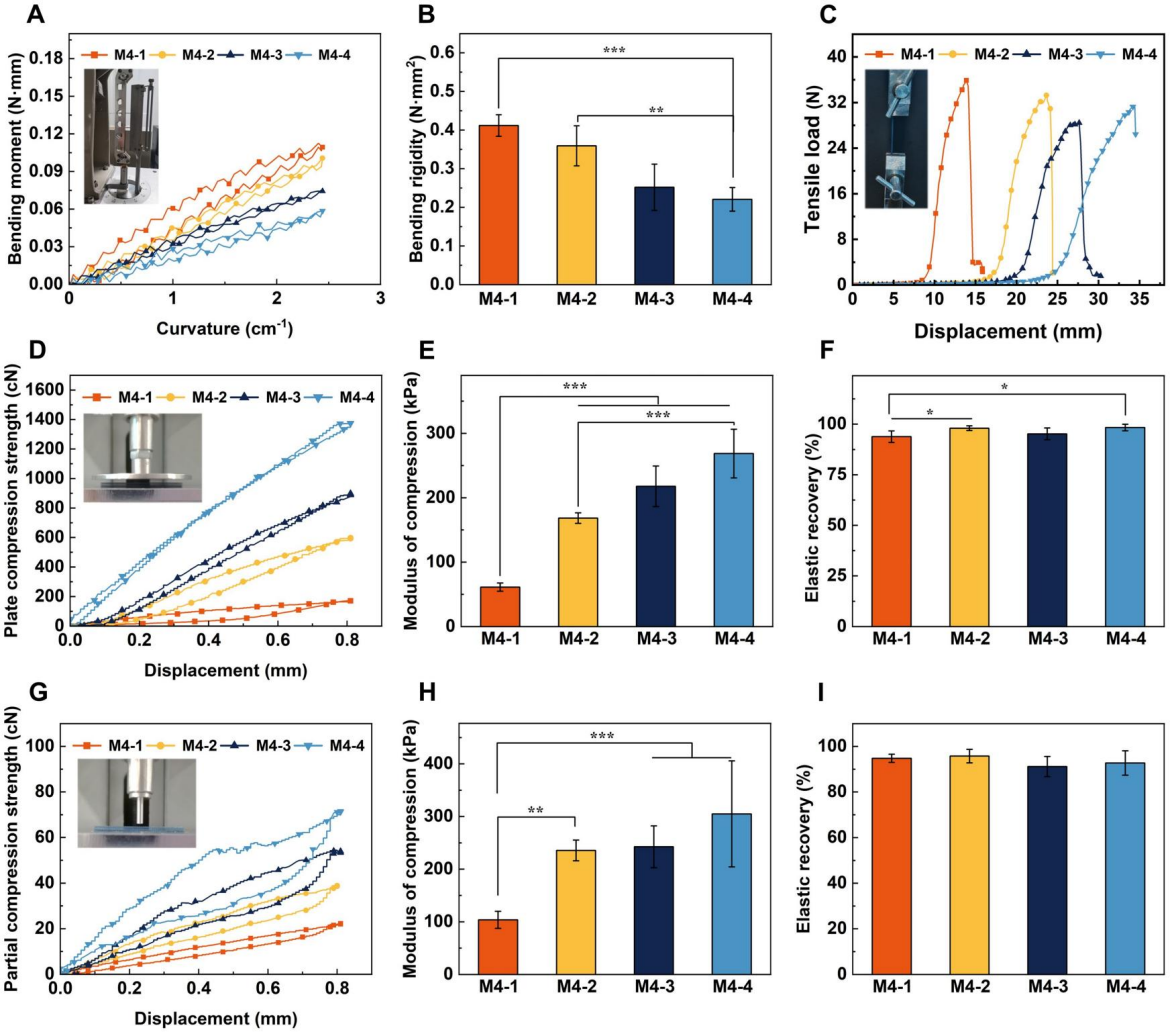
Mechanical properties of mono-layer stents group M4 (*n* = 5). (**A**) Quantitative bending test on the bending tester and bending moment curves. (**B**) Bending rigidity. (**C**) Axial stretching test diagram and tensile curves. (**D–F**) Compression-release diagram and curves, modulus and elastic recovery under plate compression. (**G–I**) Compression-release diagram and curves, modulus and elastic recovery under partial compression.

Unlike the PU stent, the angle between the wire and the axial direction (braiding angle, α) affects the flexibility and radial support of the braided stent. A high degree of flexibility means the stent is easier to bend and adapt to the anatomy of the ureter. For the qualitative bending test, two short rods with a diameter of 1.6 mm were inserted into both ends of the stent sample, and the lumen morphology at the bend was observed. All four stents in group M4 maintained excellent lumen patency after bending, whereas stents M8-1, M8-2 and M65-1 exhibited obvious flattened morphologies with occluded lumens (Figure 2). The quantitative test was finished on a semi-automatic bending tester. Group M4 exhibited the most elastic bending behavior, accompanied by the lowest bending stiffness of 0.22 ± 0.03 N·mm^2^ (Figure 3A and B). As the PPI and α increased, the bending stiffness of the group M4 stents displayed a decreasing trend in bending stiffness, and the dominant position of the wire body bending was gradually replaced by the deformation of the braided structure. Interestingly, group M65 had no obvious trend while group M8 showed an opposite increasing trend in bending stiffness. Compared to group M65 and M8, group M4 demonstrated inconspicuous plateau regions and lower energy losses (Supplementary Figure S2). These were attributed to the wire diameter-dependent stiffness and α of group M8 (44.40 ± 4.36°–66.99 ± 5.75°), group M65 (52.33 ± 4.48°–79.54 ± 6.94°) and group M4 (72.38 ± 3.43°–107.41 ± 2.27°). The increment in α corresponded to more circumferentially aligned wires, which facilitated bending deformation of the stents. Taken together, the group M4 stents demonstrated superior axial flexibility and were better able to maintain lumen patency in the bent state.

Ureteral stent fracture occurs during both the implantation process and the removal process of the stent. In most cases, stent fractures occur mainly in polymer stents [43, 44]. However, metal stents may also fracture caused by physiological activity and movement [45, 46]. The axial tensile mechanical properties of braided stents were tested by stretching 50 mm-long stents at 200 mm/min on a textiles multifunctional strength testing machine. The axial tensile curves revealed three distinct deformation phases (Supplementary Figure S3). (i) Structural reconfiguration phase: Wire networks gradually reoriented along the axial direction with braiding angle reduction and structural elongation. The tensile force remained minimal, producing near-horizontal curve profiles. (ii) Wire deformation phase: Inter-wire sliding culminated in mutual compression, transferring axial load to individual wires and the tensile force escalated rapidly, reaching peak values. (iii) Fracture Phase: The wire failed gradually or suddenly, reducing the tensile force to 0 N. In group M4 (Figure 3C), the breaking elongation escalated with increasing PPI of the stents, and this increase occurred in phase 1. Group M4 showed tensile strengths ranging from 34.27 ± 5.27 to 37.22 ± 1.77 N, which were 199.24% of the PU stent’s 17.20 ± 0.84 N.

Radial support is the vital property to support the lumen patency under compression, and to maintain the drainage function. The partial structure of the braided stent was not equivalently regarded as a homogeneous tube body, thus the plate compression and partial compression (half of ID) tests were carried out on a compression elasticity tester. The plate compression test results of three stent groups were illustrated in Supplementary Figure S4. With PPI increased, group M4 exhibited enhancements in both plate compression strength and compression modulus, and offered a good elastic recovery rate (Figure 3D–F). Stent M4-4 achieved the highest plate compression strength (1259.10 ± 167.38 cN), compression modulus (268.60 ± 37.88 kPa), and elastic recovery rate (98.34 ± 1.62%), which were comparable to those of the PU stent (plate compression strength 1270.70 ± 100.68 cN, compression modulus 527.45 ± 50.10 kPa, and elastic recovery rate 97.34 ± 1.07%). This phenomenon arose because higher PPI conferred a larger α on the stent. Consequently, greater force components were distributed along the circumferential direction while smaller components acted axially, resulting in reduced structural deformation under constrained compression displacement. Simultaneously, increased PPI elevated the number of deposited wires per unit length, thereby improving compression resistance. In contrast to groups M8 and M65, group M4 showed lower hysteresis loops, denoting low energy dissipation and excellent elastic recovery.

The partial compression test results were consistent with the plate compression observations, and all groups exhibited progressive increases in partial compression strength and modulus as PPI escalated (Supplementary Figure S5). Unlike the homogeneous block structure, the braiding structure is composed of wires and blank pores, resulting in a significant migration of wires under localized compression. Distinct hysteresis loops were detected across all groups because the compression process caused structural deformations. Compared to the partial compression strength (215.97 ± 16.02 cN), modulus (1131.50 ± 232.46 kPa), and elastic recovery rate (95.41 ± 1.62%) of the commercial PU stent, stent M4-4 presented unsatisfactory partial compression strength (59.73 ± 9.35 cN), modulus (305.00 ± 100.74 kPa), and elastic recovery rate (92.72 ± 5.32%) (Figure 3G–I). Stent M4-4 had satisfactory tensile strength, flexibility, and plate compression strength, but its partial compression strength was much lower than that of the commercial PU stent. Finite element simulations were used to analyze the structural changes in stent M4-4 during compression (Supplementary File, Figure S6), and these findings were used to guide the optimization of the stent from the mono-layer to the bi-layer structure.

### Fabrication and characterization of bi-layer braided stents

**Figure 4:**
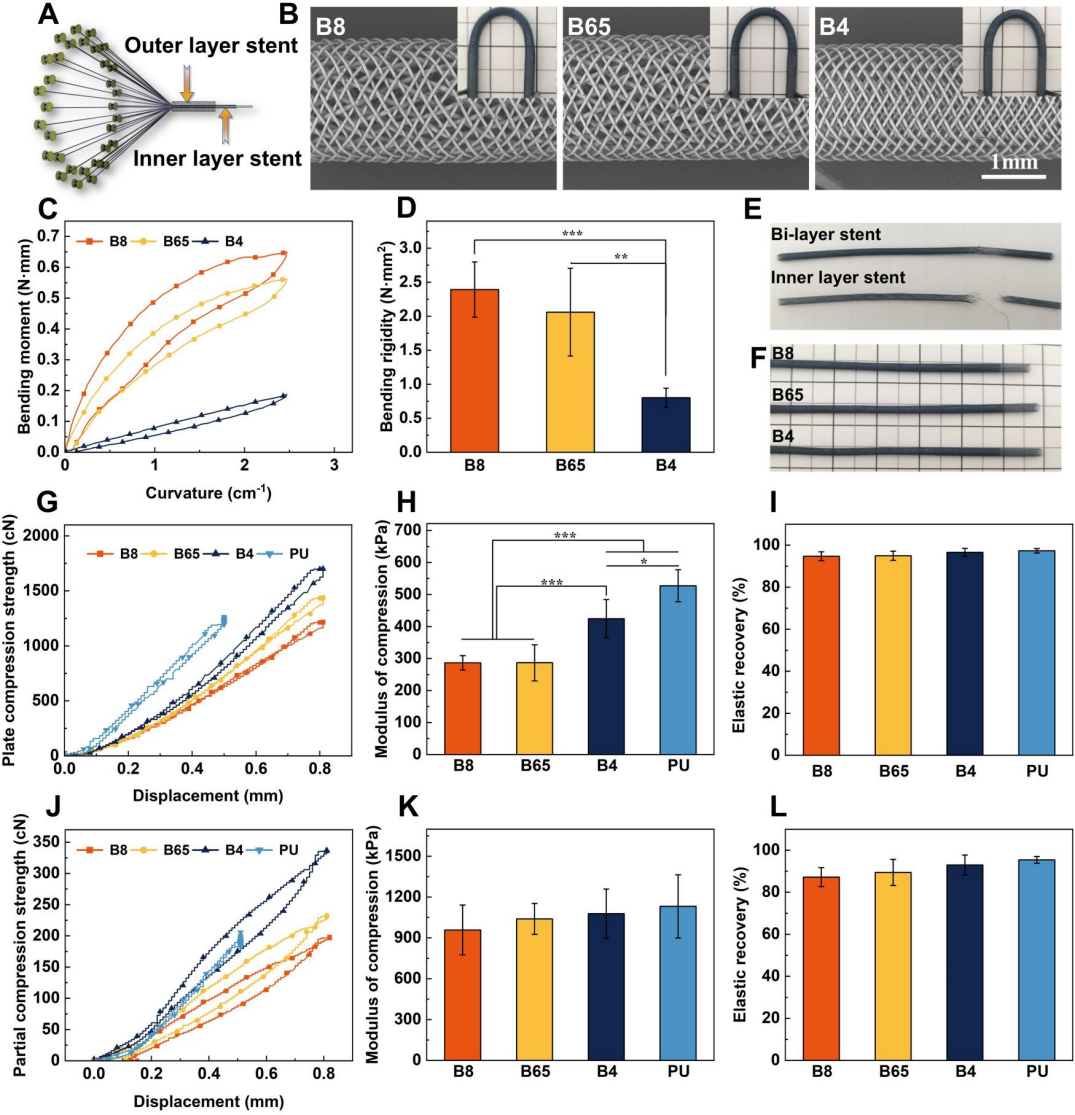
Mechanical properties of bi-layer stents (*n* = 5). (**A**) The outer layer stent of the bi-layer stent was braided around the inner layer stent. (**B**) Morphologies under SEM and qualitative bending results. The inner layer stents of B8, B65 and B4 were braided by 0.08 mm, 0.065 mm and 0.04 mm wires. (**C**) Bending moment curves. (**D**) Bending rigidity. (**E**) After stretching, the inner layer of the bi-layer stent broke before the outer layer did. (**F**) Interlayer delamination happened to bi-layer stents due to stress actions. (**G–I**) Compression-release curves, modulus and elastic recovery under plate compression. (**J–L**) Compression-release curves, modulus and elastic recovery under partial compression.

In view of the poor partial radial support of the mono-layer structure, the manufacture of bi-layer stents is a convenient method to increase the load-bearing structure and enhance the radial support. Mono-layer stents had extremely thin wall thicknesses, which provided sufficient freedom in design. We selected three mono-layer stents (from groups M8, M65 and M4, respectively) as the inner layer, and braided the outer layer with the parameters of stent M4-4 around the inner layer, forming three bi-layer stents B8, B65 and B4 (Figure 4A). The morphology of bi-layer stents B8, B65 and B4 were shown in Figure 4B. The stent OD ranged from 1.87–2.07 mm, corresponding to the PU stent (6 Fr).

All three stents maintained good lumen patency under bending conditions. However, a separation phenomenon was observed between the inner and outer layers of stent B8 (Figure 4B). The inner layer of stent B8 exhibited low PPI, leading to a slight reduction of the inner stent lumen under bending. In contrast, the outer layer had a higher PPI, resulting in minimal lumen deformation under bending. Due to the absence of bonding force between the two layers, a void formed between the inner and outer layers. In quantitative bending test, the bi-layer stent B4, with a less rigid inner layer, also exhibited lower bending stiffness and displayed a linear bending curve. Conversely, B8 and B65 demonstrated bending curves similar to those of their inner stents, indicating significant cross-sectional deformation during bending (Figure 4C).The bending stiffness of the three stents decreased sequentially: B8 > B65 > B4, with values of 2.39 ± 0.41 N·mm^2^, 2.06 ± 0.64 N·mm^2^ and 0.80 ± 0.14 N·mm^2^, respectively. This was attributed to the variation in bending stiffness of the inner layers (Figure 4D).

The tensile stages of the three bi-layer stents were similar to those of mono-layer stents (Supplementary Figure S7). The distinct characteristic lay in the presence of double tensile fracture peaks for two layers, respectively. For stents B8 and B65, the significant difference in braiding angles between their inner and outer layers resulted in asynchronous deformation stages during stretching. Although stent B4 shared identical braiding parameters for two layers, the actual increase in mandrel size during outer layer fabrication caused the practical α of the outer layer to exceed that of the inner layer. For stent B4, this discrepancy resulted in the fracture of the inner layer first, followed by that of the outer layer (Figure 4E). Moreover, after repeated tests, the asynchronous deformation and recovery between the two layers induced interlayer delamination, resulting in irreversible deformation and compromised structural recovery (Figure 4F).

All three bi-layer stents exhibited significantly enhanced plate and partial compression forces. The plate compression and recovery curves nearly overlap in the region close to the zero position and low energy dissipation (Figure 4G), indicating the excellent elastic recovery (Figure 4I). Specifically, stent B4 achieved remarkable plate compression force of 1718.13 ± 81.68 cN, surpassing that of PU stent (1270.70 ± 100.68 cN). Meanwhile, stent B4 displayed a significantly higher modulus of 424.48 ± 59.78 kPa than 268.60 ± 37.88 kPa of stent M4-4, approaching the value of PU stent (527.45 ± 50.10 kPa) (Figure 4H). Notably, stent B4 achieved remarkable partial compression forces of 282.99 ± 76.16 cN, outperforming the PU stent (215.97 ± 16.02 cN). At the same compression distance, stent B4 still had a higher partial compression strength than the PU stent (Figure 4J). Partial compression modulus of bi-layer stent B4 reached 1077.83 ± 180.71 kPa (Figure 4K), which was not only much higher than that of mono-layer M4-4 (305.00 ± 100.74 kPa) but also approached PU stent benchmarks (1131.50 ± 232.46 kPa), while simultaneously demonstrating the highest elastic recovery rate (Figure 4L). Furthermore, by simulating the deformation of stent B4 during compression using finite element analysis, the structural reasons for the increased compressive strength of the bi-layer stent were explained.

The development of three bi-layer braided stents revealed that stent B4 achieved the best radial support and flexibility with a thinner wall. The braided bi-layer structure significantly improved the mechanical properties of the stent, especially compensated for the deficiency of partial compression strength of the braided mono-layer structure. Whereas, the lack of interlayer bonding led to delamination and irreversible deformation of the stents after stress deformation. Thus, it was necessary to enhance the interlayer bonding.

### Fabrication and characterization of interlocked bi-layer braided stents

**Figure 5:**
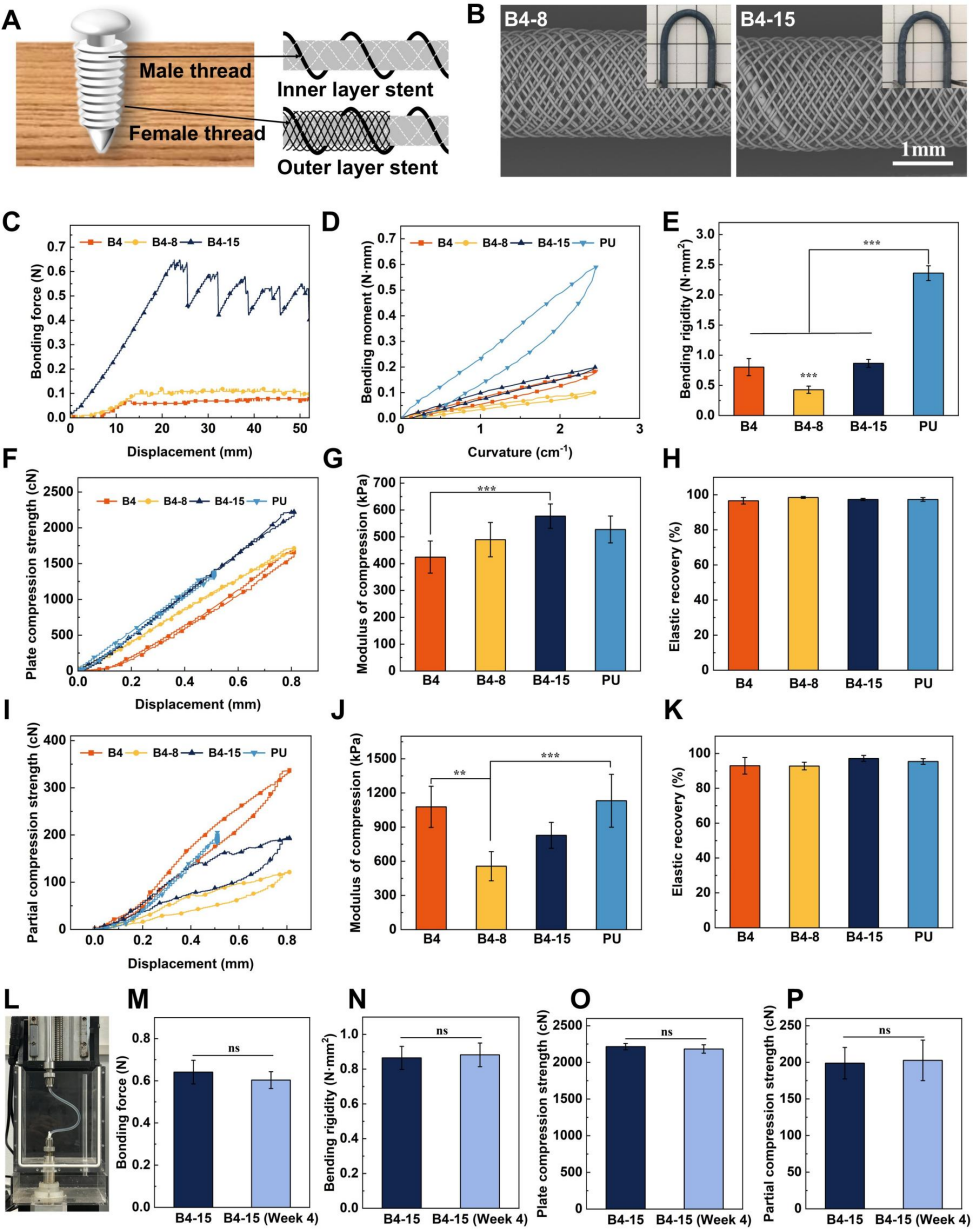
Structural design and mechanical properties of interlocked bi-layer stents (*n* = 5). (**A**) The structure of the interlocked bi-layer stent was inspired by engagement of threads, the inner layer corresponds to the male thread and outer layer corresponds to the female thread. (**B**) Morphologies under SEM and qualitative bending results. A thick wire of 0.08 mm and 0.15 mm was braided within the inner layer of stent B4-8 and stent B4-15 respectively. (**C**) Bonding force between inner and outer layer. (**D**) Bending moment curves. (**E**) Bending rigidity. (**F–H**) Compression-release curves, modulus and elastic recovery under plate compression. (**I–K**) Compression-release curves, modulus and elastic recovery under partial compression. (**L**) Fatigue test conducted on custom-built tensile and torsion fatigue testing device. (**M–P**) Property comparisons of stent B4-15 before and after four-week fatigue test, which included interlayer bonding force, bending rigidity, plate and partial compression strength.

Inspired by the thread engagement (male thread and female thread), we expected to build a male thread structure in the inner layer stent. Further, the outer layer stent braided with this male thread structure as a mandrel then spontaneously formed a matching female thread structure, ultimately interlocking the inner and outer layers (Figure 5A). Based on the bi-layer stent B4, a single wire in the inner layer was replaced with a thicker NiTi wire (0.08 mm or 0.15 mm) to form a male thread structure. Subsequently, the outer layer stent was shape-adaptively braided to form the interlocked bi-layer stents B4-8 and B4-15. The OD of stent B4, B4-8 and B4-15 were 1.87 ± 0.03 mm, 1.85 ± 0.03 mm and 1.91 ± 0.03 mm, respectively. Meanwhile, the wall thicknesses were 0.27 ± 0.03 mm, 0.25 ± 0.03 mm and 0.31 ± 0.03 mm, all of which were thinner than that of the commercial stent (0.39 ± 0.01 mm). The structure of stents B4-8 and B4-15 were uniform (Figure 5B). It is worth noting that there was only one thick wire in the interlocked bi-layer stent, which would lead to significant wall discrepancy. We recorded the maximum thickness (near the thick wire) of the cross-section as the wall thickness.

To verify the engagement of threads resulting from the thicker wire, we stretched the stent axially by holding the inner layer and outer layer separately. The interlayer bonding forces of the three stents were 8.33 ± 1.71 cN, 14.30 ± 2.60 cN and 64.10 ± 5.62 cN, respectively (Figure 5C). The tensile curves of stents B4 and B4-8 were generally similar, while for stent B4-15, the inner and outer layers repeated a peak pattern every time they moved relatively to each other by a pitch distance. This indicated that the 0.15 mm wire in stent B4-15 played a significant role in interlocking the structure. The tensile curves of stents B4, B4-8 and B4-15 were similar, especially in the initial period when they were quite flat, and then the slope rose sharply until the wire broke (Supplementary Figure S8). The tensile strengths of the three stents were 34.97 ± 4.99 N, 39.50 ± 9.94 N and 38.44 ± 1.83 N, respectively, with no significant differences, all of which were significantly higher than that of PU stent (17.20 ± 0.84 N). The elongation at break for the three stents were 68.03 ± 3.83%, 68.45 ± 1.15% and 72.88 ± 8.89%, respectively. NiTi stents exhibited superior corrosion resistance and a higher breaking force, which ensured safe and reliable stent removal. Due to the introduction of the thicker wire, the bending stiffness and radial support of the stent were also enhanced. Similar to stent B4, both stents B4-8 and B4-15 maintained lumen patency under bending (Figure 5B), and both had lower bending moments than the PU stent (Figure 5D). The bending stiffness of stents B4, B4-8, B4-15 and PU stent were 0.80 ± 0.14 N·mm^2^, 0.43 ± 0.06 N·mm^2^, 0.87 ± 0.07 N·mm^2^ and 2.36 ± 0.12 N·mm^2^, respectively. Among them, stent B4-15 showed a 63.14% reduction in bending stiffness compared to the PU stent, denoting its superior flexibility (Figure 5E).

Compared to the PU stent and stent B4, both stents B4-8 and B4-15 exhibited enhanced plate compression strength with the diameter of the thick wire increased (Figure 5F). Stent B4-15 demonstrated the highest plate compression strength of 2215.44 ± 42.87 cN and plate compression modulus of 577.02 ± 45.31 kPa, as well as excellent elastic recovery when maintaining 50% lumen patency (Figure 5G and H), which was closely related to the high stiffness of the 0.15 mm wire and the stronger interlayer bonding force. The partial compression strengths of stents B4, B4-8, B4-15 and PU stent were 282.99 ± 76.16 cN, 123.69 ± 3.06 cN, 198.91 ± 21.48 cN and 215.97 ± 16.02 cN, respectively. After significant partial compression, these stents exhibited an energy dissipation in recovery (Figure 5I). Stents B4-8 and B4-15 displayed lower moduli than that of stent B4 (Figure 5J), as well as excellent elastic recovery (Figure 5K). On the one hand, the introduction of the thick wire undoubtedly improved mechanical properties. On the other hand, the thick wire occupied more space for braiding, resulting in uneven distribution of the other wires, which in turn led to a loss of partial compression strength.

During implantation, the ureteral stent is subjected to repeated physiological and mechanical stresses, such as bending. We conducted a four-week fatigue test on stent B4-15 using a custom-built tensile and torsion fatigue testing device (Figure 5L), and then compared the key mechanical properties of the post-fatigued B4-15 (Week 4) with those of the pre-fatigued B4-15. Statistical analysis indicated no significant differences (*P* > 0.05) in any of the critical mechanical indices between B4-15 (Week 4) and B4-15. Specifically, the interlayer bonding force remained stable (60.33 ± 4.00 cN), confirming that the thread-inspired interlocking strategy effectively prevented delamination or structural slippage even after prolonged dynamic deformation (Figure 5M). The stent maintained both its plate and partial compression strengths (2182.79 ± 57.87 cN and 202.65 ± 27.68 respectively) (Figure 5N and O) with excellent elastic recovery (97.50 ± 2.17% and 94.29 ± 5.16%) (Supplementary Figure S9), illustrating its superior stability of radial support. Moreover, the stable bending rigidity (0.88 ± 0.07 N·mm^2^) demonstrated that the NiTi wire network effectively preserved its structural flexibility.

Collectively, we ultimately designed two types of interlocked bi-layer stents, and compared them with the original bi-layer stent B4 and PU stent. Stent B4-15 addressed the issue of insufficient interlayer bonding force in bi-layer stents, demonstrated superior radial support compared to the PU stent, and exhibited thinner wall and enhanced flexibility. Therefore, it was selected as the optimal structural design.

### Drainage property of interlocked bi-layer braided stents

Drainage is the most essential function of of a ureteral stent, referring to the ability to guide urine flow in the direction of kidney-ureter-bladder. Ureteral stents provide immediate pain relief by decreasing renal pelvic pressure [47]. Inefficient drainage can lead to urine retention and recurring increase in renal pelvic pressure, while facilitating further occlusion of the stent [48]. Ureteral stenosis and compression of tumor can in turn weaken the drainage performance of ureteral stents. In accordance with Poiseuille’s law, flow rate is positively correlated to the fourth power of the diameter of the tube (Equation (1)). Therefore, it is necessary to measure the drainage property of ureteral stents at different degrees of lumen loss and relative lower renal pelvic pressure.


(1) Q= πΔPD4128μL

Where *Q* represents the flow rate per unit time, *ΔP* represents the pressure difference between the two ends of the tube, *μ* represents the urine viscosity, *L* represents the effective drainage length of the stent, and *D* represents the ID of the stent.

**Figure 6:**
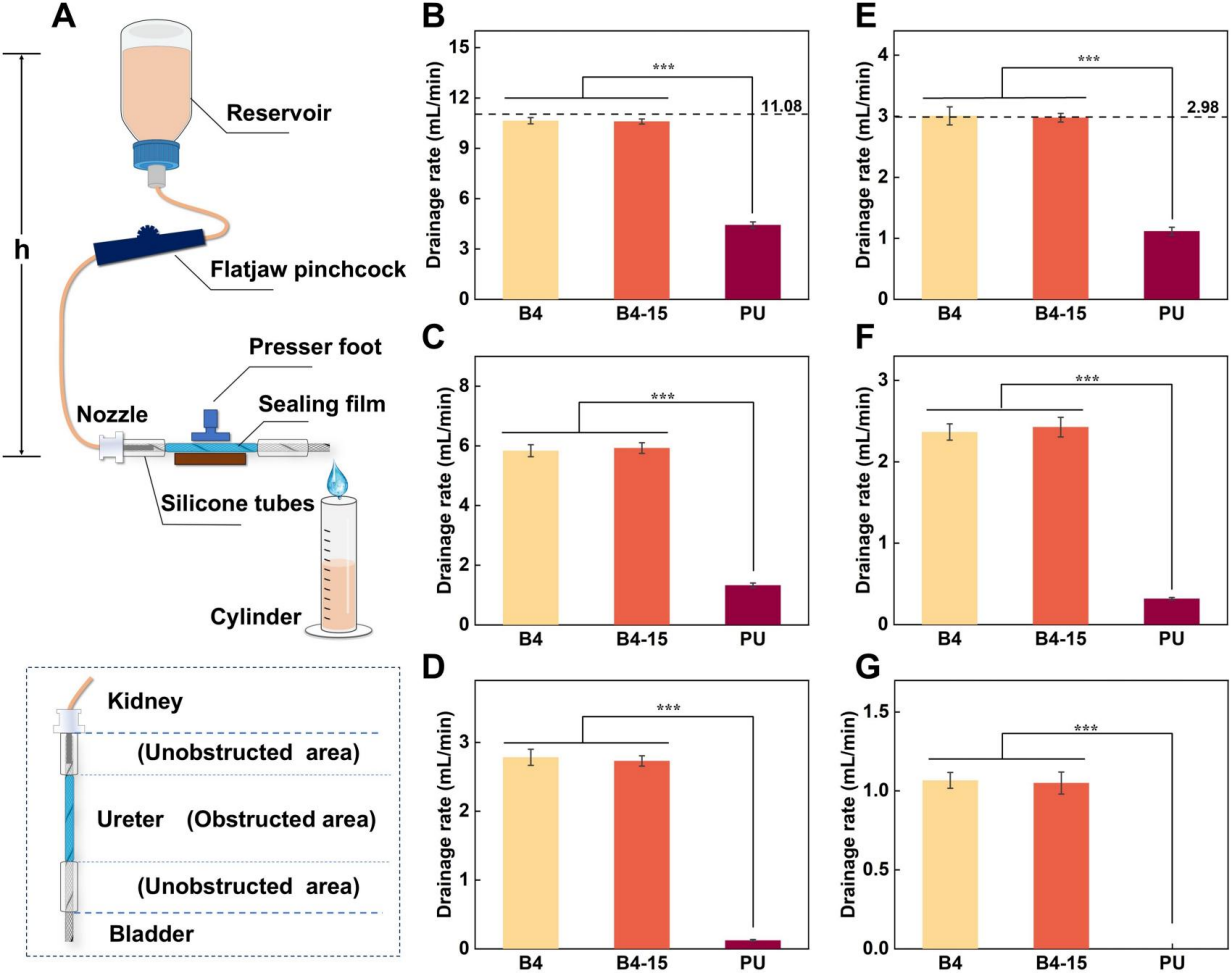
Drainage test (*n* = 3). (**A**) Schematic diagram of drainage test system. (**B–D**) Drainage properties of stent B4, stent B4-15 and the commercial PU stent compressed by 0 mm, 0.8 mm and 1.0 mm, respectively under 10 cm H_2_O column pressure. (**E–G**) Drainage properties of stent B4, stent B4-15 and the commercial PU stent compressed by 0 mm, 0.8 mm and 1.0 mm, respectively under 4 cm H_2_O column pressure.

A drainage test system was constructed including four parts: (i) the kidney simulation part with a reservoir bottle and a nozzle, (ii) the unobstructed-ureter part with the ends of stent inserted in two thin-wall silicone tubes, (iii) the obstructed-ureter part with the stent sealed by a sealing film, (iv) the bladder simulation part with the stent exposed (Figure 6A). To simulate the loss of ureteral stent lumen, the compression of constant distances were exerted on the obstructed ureter part. In previous reports, 20 cm H_2_O was often selected as the simulation pressure for drainage tests, which was generally set based on the physiological value of renal pelvis pressure (below 20 cm H_2_O) [47, 49]. What we need to verify is whether the ureteral stent can provide a valid drainage while keeping the renal pelvis pressure as far away from this critical value as possible.

In order to verify the preservative effect of the stent on the physiological drainage rate of the ureter, we adjusted the height difference (*h*) to obtain a high initial flow rate of 11.08 ± 0.54 mL/min (10 cm H_2_O), which was close to the physiological drainage rate from the renal pelvis to the ureter (8 mL/min). At high initial flow rate, when the lumen of the stent was absolutely patent (0 mm compression), the drainage rates of stent B4 (10.64 ± 0.19 mL/min) and stent B4-15 (10.60 ± 0.15 mL/min) were very similar to the initial flow rate (11.08 ± 0.54 mL/min) and much higher than that of the PU stent (4.44 ± 0.18 mL/min). (Figure 6B). Under a compression of 0.8 mm (half of ID), stents B4 and B4-15 demonstrated higher drainage rates than the PU stent (Figure 6C). When the compression distance came to 1.0 mm (simulating a severe obstruction), stents B4 and B4-15 were still capable of maintaining > 2.7 mL/min (24.37% of the initial flow rate). Whereas, only 0.12 ± 0.01 mL/min (0.01% of the initial flow rate) was retained by the PU stent (Figure 6D).

When luminal loss occurs, the resistance to urine drainage inevitably increases. As the urine accumulates, the renal pelvic resting pressure rises to values exceeding the normal range. The test was further conducted applying a low initial flow rate of 2.98 ± 0.04 mL/min generated by a pressure of 4 cm H_2_O, which is close to the renal pelvic resting pressure. The results at low flow rate were similar to those at high-flow rate. When the stent was uncompressed, stents B4 and B4-15 exhibited drainage rates (3.01 ± 0.15 mL/min and 2.98 ± 0.07 mL/min) closely matching the initial flow rate (2.98 ± 0.04 mL/min), demonstrating efficient lumen patency. In contrast, the PU stent showed 1.12 ± 0.07 mL/min (37.58% of the initial flow rate) (Figure 6E). Under 0.8 mm compression, stents B4 and B4-15 retained substantial drainage capacity (2.37 ± 0.10 mL/min, 2.43 ± 0.12 mL/min), maintaining over 79.53% of drainage. While the PU stent declined sharply to 0.32 ± 0.02 mL/min, losing 71% of its drainage rate (Figure 6F). Under 1.0 mm compression, stents B4 and B4-15 preserved over 1 mL/min (33.56% of the initial flow rate), while the PU stent lost entire drainage performance owing to excessive lumen loss (Figure 6G).

All the results (Supplementary Table S6) showed a significant positive correlation between stent ID and drainage rate. Stent B4-15 had a 0.31 ± 0.03 mm wall thickness and achieved a 29.03% increase in ID compared to the PU stent. The introduction of the thick wire did not change its drainage performance. The larger internal diameter and excellent mechanical properties allowed stent B4-15 to maintain higher drainage rate at all compression levels and could ensure timely drainage while maintaining low pelvis pressure.

### Retention force evaluation of interlocked bi-layer braided stents

**Figure 7:**
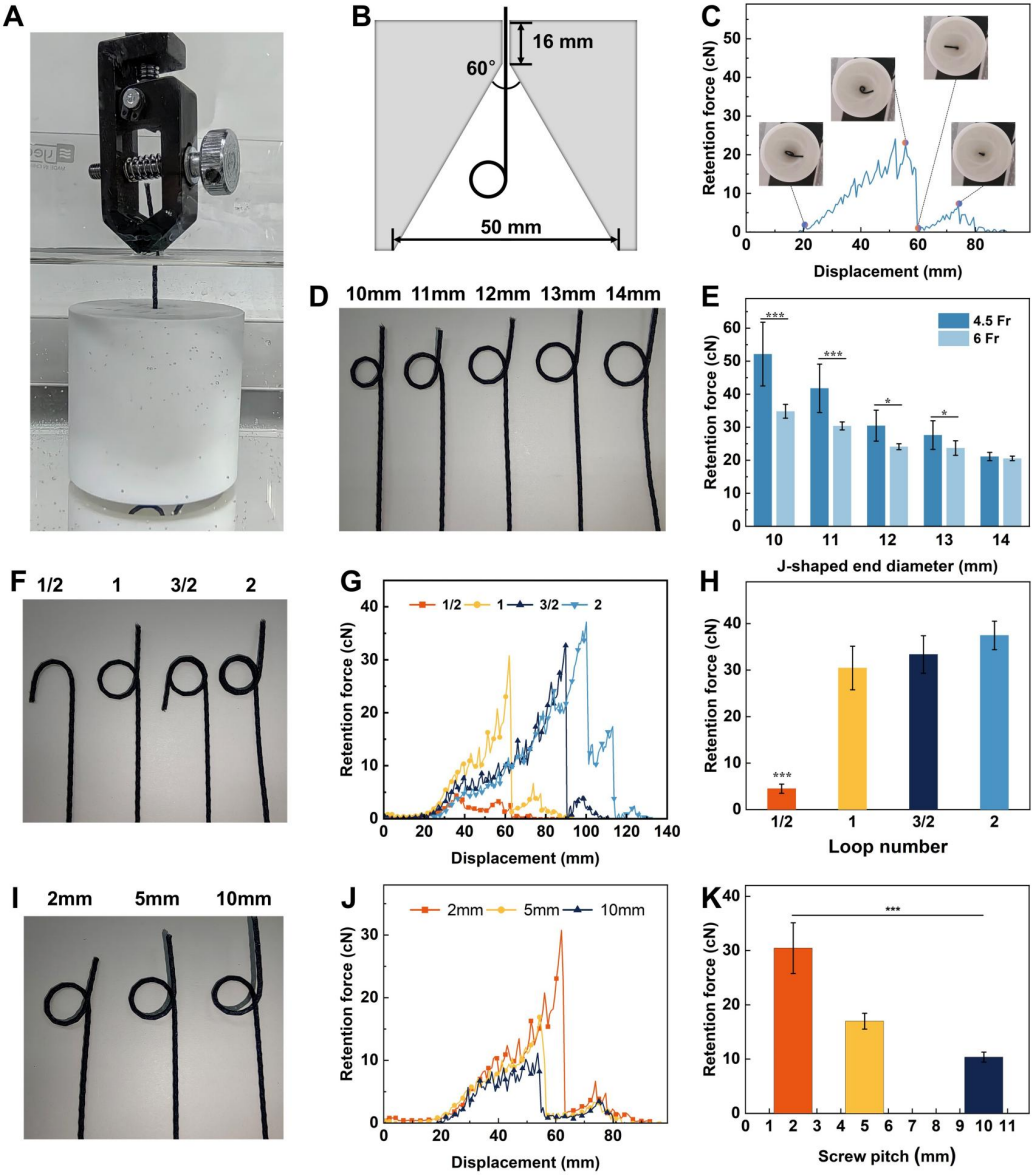
Anti-migration test (*n* = 3). (**A**) Test method: the J-shaped end was pulled out of the PTFE mold. (**B**) Size parameters of the PTFE mold. (C) Test curve and corresponding morphology of J-shaped end with typical parameter (J-shaped end diameter 12 mm, one loop, pitch 2 mm). (**D**) Photos of J-shaped ends with different diameters. (**E**) Retention forces of 4.5 Fr and 6 Fr stents with J-shaped end diameters 10–14 mm. (**F–H**) Photos, test curves and retention forces of J-shaped ends with different loop numbers. (**I–K**) Photos, test curves and retention forces of J-shaped ends with different pitches.

Currently, the dominant clinical products are double-J (D-J) stents, and both ends of the D-J stent are moulded into a J-shape to secure them within the kidney and bladder, respectively. The retention force refers to the anti-migration ability of the stent after implantation. To the best of our knowledge, the relationship between the structural parameters of the J-shaped end and the retention force of the stent has not yet been investigated. The braided stent has a different structure from the extruded PU stent, and it is necessary to investigate the structure–function relationship between the J-shaped end structure and the retention force. The J shaped end was inserted in the PTFE mold followed by the other end fixed on fixture, and pulled out the J shaped end from the mold at a speed of 500 mm/min (Figure 7A). Based on the stent B4-15, we further adjusted the stent caliber, followed by the diameter, number and pitch of the J-shaped end to explore the effects of structural parameters on the retention force.

Figure 7C showed the morphological changes in the end loop of stent B4-15 during the retention force test. In the initial stage, the tensile curve closely followed the horizontal axis. This was due to the deformation of the braided structure under low tension. As stretching continued, the curve fluctuated upward until a peak appeared when the main body of the end loop passed through the funnel-shaped opening. In the final stage, only the curved end loop remained and gradually passed through the funnel-shaped opening, resulting in a lower secondary peak. Therefore, the first peak value was taken as the retention force. Referring to the structure of PU stent end, a braided stent with specification of 6 Fr, a diameter of 12 mm, one loop and a pitch of 2 mm, was selected as the reference sample. The retention force of stent B4-15 was measured to be 24.08 ± 0.98 cN, which was close to 27.83 ± 1.33 cN of the PU stent, proving that stent B4-15 met the clinical requirement for retention force with the same caliber.

When operating with stents of different sizes, the stents conveyed different tactile sensations, which caught our attention. Thus, we investigated the stents B4-15 with different calibers J-shaped end diameter (Figure 7D). With the increase in end diameter, the retention force decreased. The 4.5 Fr stent exhibited higher retention forces than those of the 6 Fr stent under the same conditions (Figure 7E). The retention force was primarily generated by the resistance of the curled J-shaped end to straightening during the tensile process, which was directly influenced by the bending stiffness of the hollow tube body. The relationship between the tube diameter (ID and OD), material elastic modulus (*E*), and bending stiffness (*EI*) is expressed as


(2) EI=E·I=E· π(D04-Di4)64

Where *I* is the inertia moment of the cross-sectional area, *D_0_* is the OD of the tube, *D_i_* is the ID of the tube.

The difference between the OD and ID of a braided stent was always equal to twice the wall thickness. Simply referring to Equation (2), the larger the stent caliber, the greater the bending stiffness, which was inconsistent with the actual results. Because a braided stent is not a simple hollow tube but also has a braided structure formed by the interwinding of wires. During reversible elastic bending deformation, changes in the braided structure replaced the strain of ordinary materials. Therefore, when analyzing the structure–property relationship, in addition to analyzing the shape of the J-shaped end and the tube structure, the influence of the braided structure should also be considered. For the stent with larger caliber, the wires are more sparsely distributed in the circumferential direction. When subjected to forces, the mutual constraint and force transmission between the wires are relatively weaker, making it easier for the wire displacement to occur, thereby reducing the retention force. This situation could also be understood as the sparse distribution of wires reducing the conceptual tube elastic modulus.

Based on physiological structure, an increase in the diameter of the J-shaped end helps the stent to be more easily fixed in the upper urinary tract. However, this does not mean the retention force will be enhanced accordingly. A larger end diameter corresponds to a larger radius of curvature (*ρ*). The end with the larger diameter is also formed from a longer section of the stent, and we considered the braided structure of the J-shaped end with different diameters to be consistent, which suggested that the *EI* could be considered a constant. According to Equation (3), for a larger end diameter, bending moment (*M*) required to straighten a J-shaped end is smaller. According to Equation (4), when the variable is only the end diameter, the deformation energy (*U*) required for this process is related to the M and the beam length (*L*). According to Equation (5), *L* is directly dependent on *ρ*. Based on the above formulas, *U* is positively correlated with 1/*ρ*. Therefore, the deformation energy of J-shaped end with a larger end diameter is lower, making it easier to be straightened.


(3) 1ρMEI

(4) U=M2L2EI

(5) L=2πρ

The more loops there are, the longer the tube after straightening will be, which further increases the difficulty of stent insertion and removal; moreover, introduces more foreign matter. Therefore, it is necessary to determine an appropriate range. Considering the negative correlation between retention force and tube diameter, 4.5 Fr stents were selected for further structural exploration to reduce the interference of the artificial error and the instrument error. We set four different loop numbers for the J-shaped end: 1/2, 1, 3/2 and 2 loops (Figure 7F). The morphological changes and retention forces of stents with different loop numbers varied during the stretching process. The stent with 1/2 loop had an incomplete end loop, resulting in an incomplete test curve which accompanied retention force of only 4.49 ± 0.99 cN. The retention force of the other three loop numbers were 30.45 ± 4.68 cN, 33.35 ± 4.03 cN and 37.46 ± 3.07 cN, respectively, and their curves were similar to those of the reference sample (Figure 7G and H). When the loop number increased from 1/2 to 1, the retention force significantly increased. During the process where the loop count increased from 1 to 2, the increase in retention force was extremely small. This demonstrated the crucial importance of a complete end loop structure for maintaining strong retention force. Furthermore, we set up the J-shaped end structure with three gradients of pitch 2, 5 and 10 mm (Figure 7I). Figure 7J showed that the three parameters of pitch did not change the morphology of the mechanical curves obviously in the tensile process. The retention force were 30.45 ± 4.68 cN, 16.99 ± 1.47 cN, and 10.36 ± 0.93 cN, respectively (Figure 7K), indicating excessive pitch could not yield a good stable support condition with the funnel-shaped opening. Thus, the J-shaped end should be designed with a smaller pitch.

## Conclusion

This research developed a novel interlocked bi-layer NiTi braided stent that effectively reconciled the conflicting requirements including low wall thickness and excellent mechanical properties while maintaining the commonly used diameter. By optimizing the wire diameter and PPI, a mono-layer stent M4-4 with favorable performances was first optimized. To further enhance the stent’s radial support, a bi-layer stent B4 was developed based on stent M4-4, which exhibited superior radial support and flexibility compared to the commercial PU stent, but suffered from inter-layer instability. The pivotal innovation was the integration of a thick wire into the inner layer stent, creating a thread-engagement structure. The optimized interlocked bi-layer stent B4-15 exhibited robust interlayer bonding (64.10 ± 5.62 cN), eliminating delamination and ensuring structural integrity under compressive physiological stresses.

Stent B4-15 provided 174.38% higher plate compression strength and 63.14% lower bending stiffness than those of the commercial PU stent, while its ultra-thin wall (0.31 mm) enabled a 29.03% larger lumen. Crucially, the stent preserved significant drainage capacity (33.56%) under severe compression, moreover, its structural stability and critical mechanical properties were maintained throughout the four-week fatigue test (*P* > 0.05). As a result, the concept of interlocked bi-layer braiding offered a scalable design for the next-generation ureteral stents and other minimally invasive tubular implants.

Despite the mechanical advantages demonstrated in this study, the site-specific biocompatibility and encrustation formation of the NiTi braided architecture remain to be evaluated under physiological conditions. Although NiTi is a widely used material in urology with proven biocompatibility and physicochemical stability, further validation of the host tissue response to this specific bi-layer braided structure is required. Long-term stability should be investigated using *in vivo* animal models to assess mineral deposition and the effects of urinary corrosion on the structural integrity of the ultra-thin wires. Future studies will focus on these biological assessments and the development of functional surface modifications to improve the overall clinical performance of the stent.

## Supplementary Material

rbag130_Supplementary_Data

## Data Availability

The data are available from the corresponding author on reasonable request.
