# Supplementary material for: Design of novel interlocked bi-layer NiTi braided stent with ultra-thin walls for urinary tract obstruction treatment
Source: Regen Biomater. 2026 Jun 15;13:rbag130. doi: 10.1093/rb/rbag130 (PMC13331286; doi:10.1093/rb/rbag130)
Supplement: rbag130_Supplementary_Data [file rbag130_supplementary_data.docx]

**Supplementary Materials**

**Design of Novel Interlocked Bi-Layer NiTi Braided Stent with Ultra-Thin Walls for Urinary Tract Obstruction Treatment**

Wenshuo Zhao,^1,2,3,#^ Jianjin Wang,^4,#^ Chenglong Yu,^5^ Yuecheng Yu,^1,2^ Xiaoli Liu,^1^^,2^ Jie Qiao,^1^ Sanieng Lei,^4^ Dong Cao,^5^ Wenping Jian,^1^ Langda Xu,^1^ Fan Zhao,^1,3,*^ Jing Lin,^1,2^ Chaojing Li,^1,3^ Fujun Wang,^1,3^ Aijun Guo,^5^ Lu Wang^1,2,^[[1]](#footnote-1)^*^


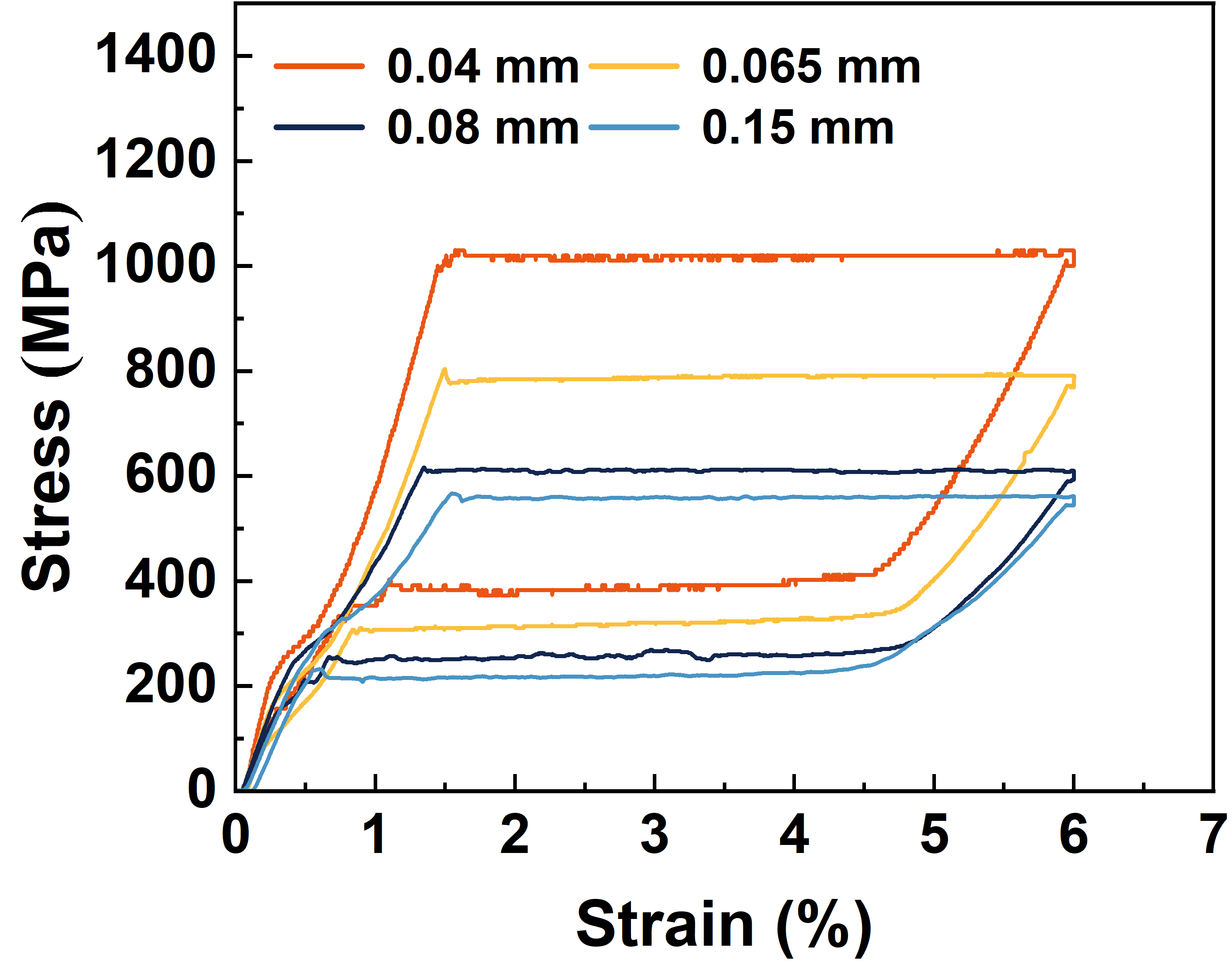


**Figure S1.** Superelastic stress–strain curves of NiTi wires with diameters of 0.04 mm, 0.065 mm, 0.08 mm and 0.15 mm. All heat-treated wires exhibited distinctive upper and lower plateau stresses and low residual strains, demonstrating stable non-linear superelastic behaviors.


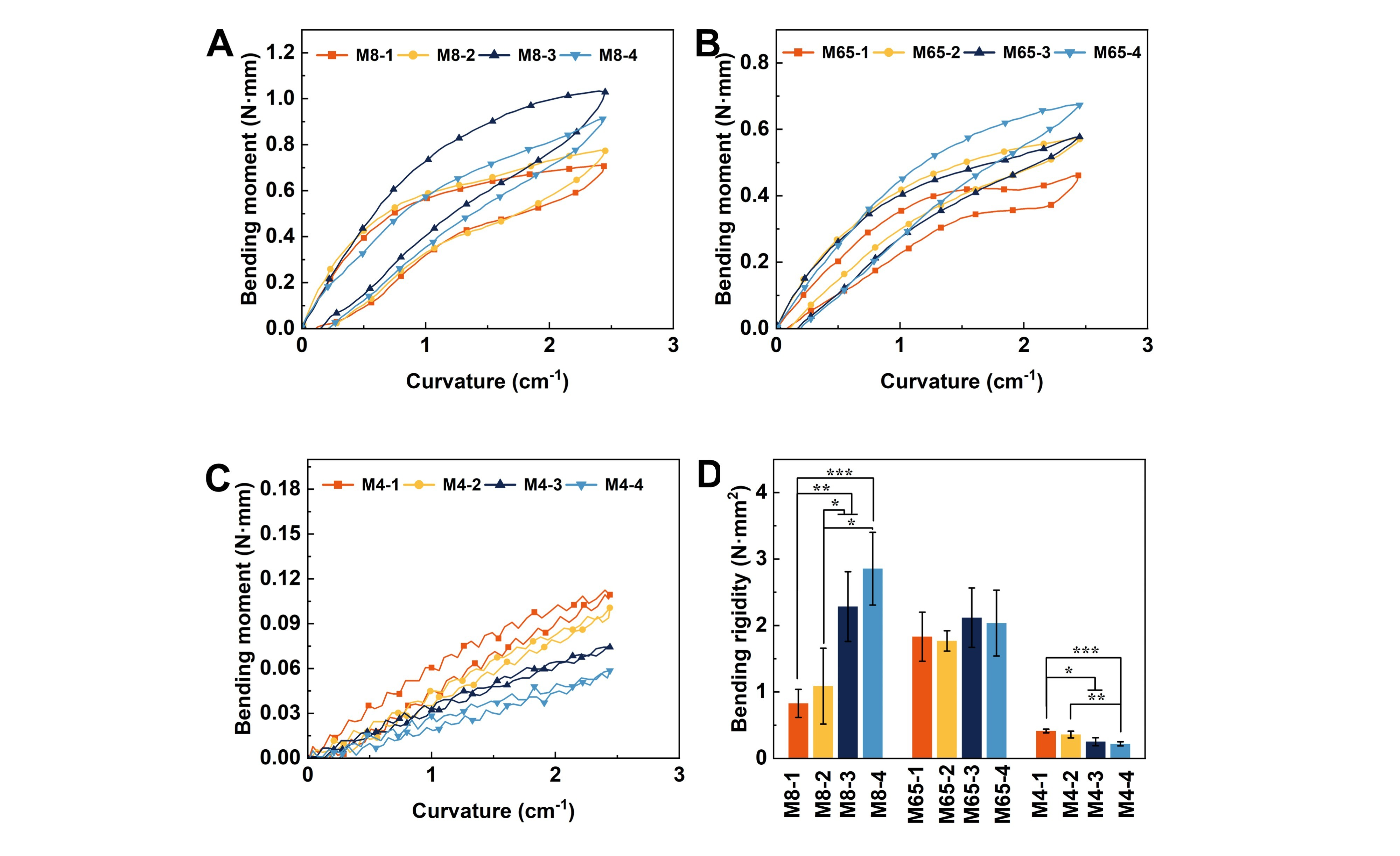


**Figure S2.** Bending property of mono-layer stents (n = 5). (A-C) Bending moment curves of group M8, group M65 and group M4. (D) Bending rigidity of group M8, M65 and M4.


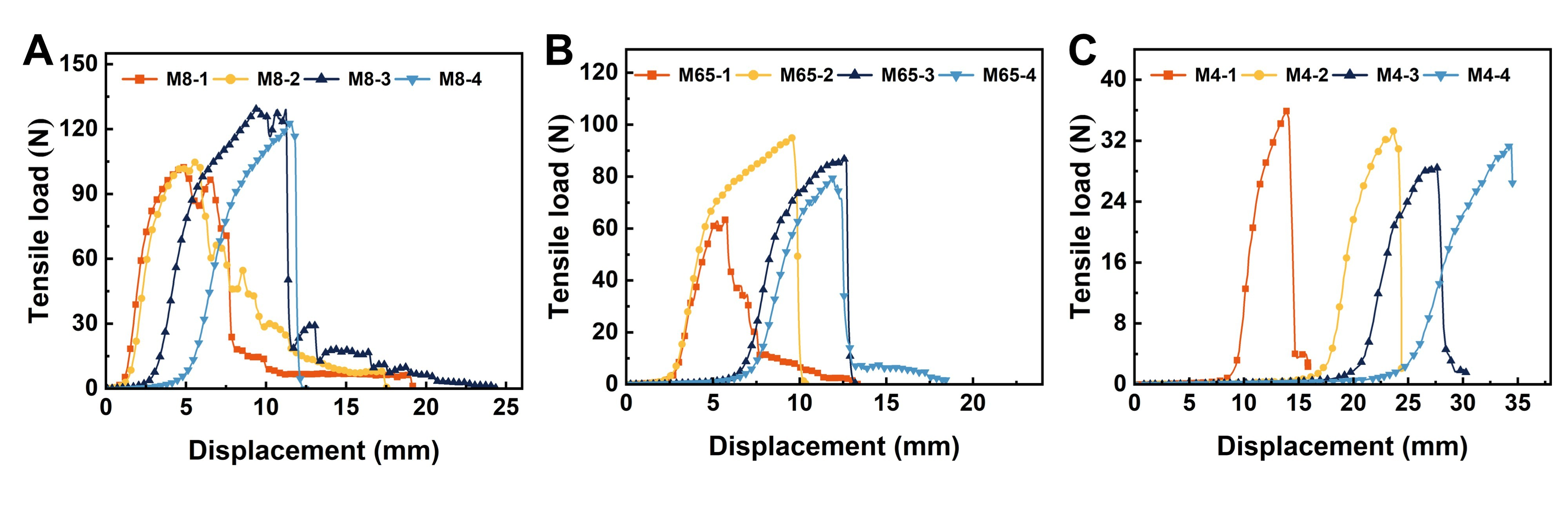


**Figure S3.** Axial tensile curves of mono-layer stents. (A) Group M8. (B) Group M65. (C) Group M4.


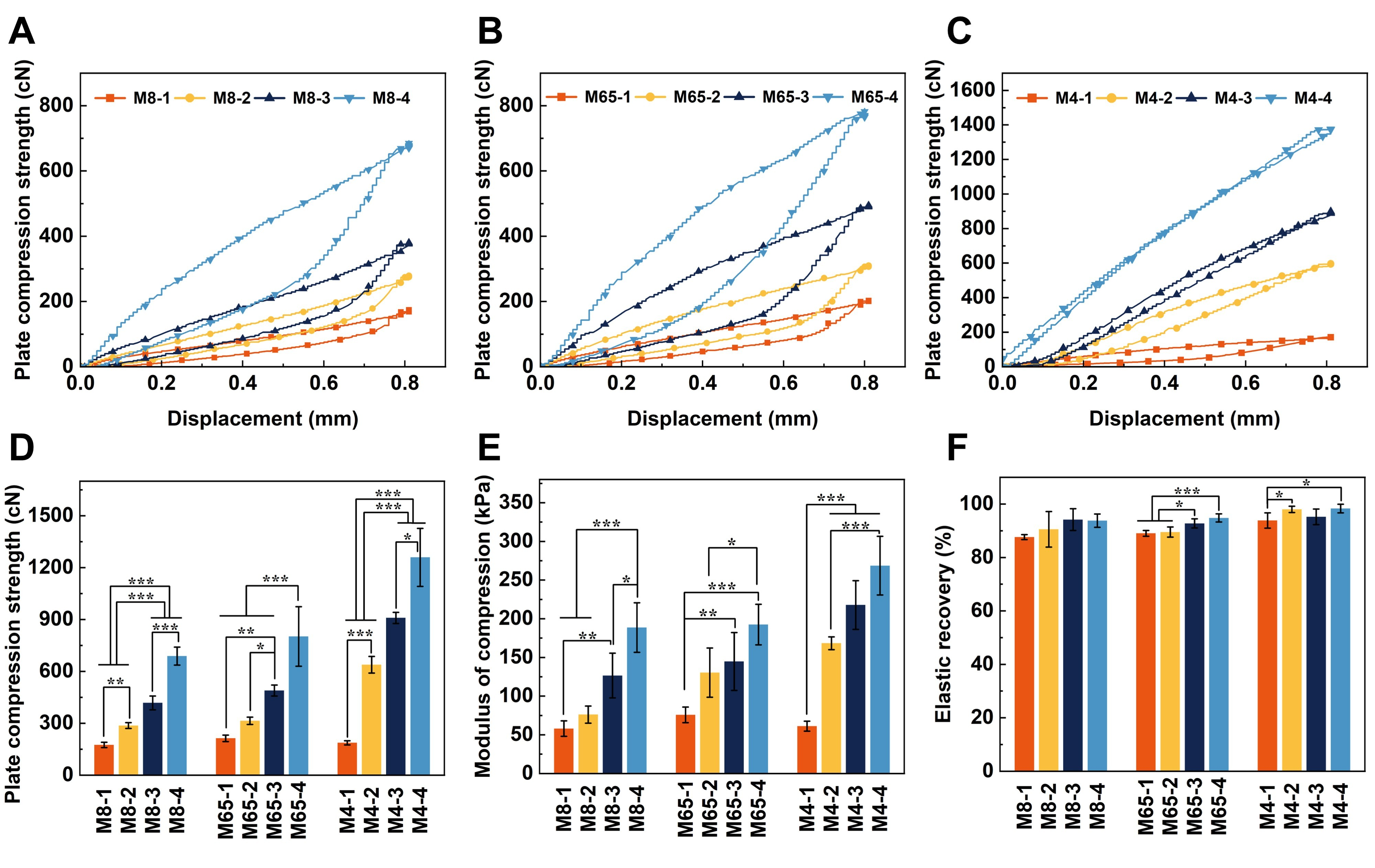


**Figure S4.** Plate compression property of mono-layer stents (n = 5). (A-C) Compression-release curves of group M8, M65 and M4. (D-F) Compression strength, modulus and elastic recovery of mono-layer stents.


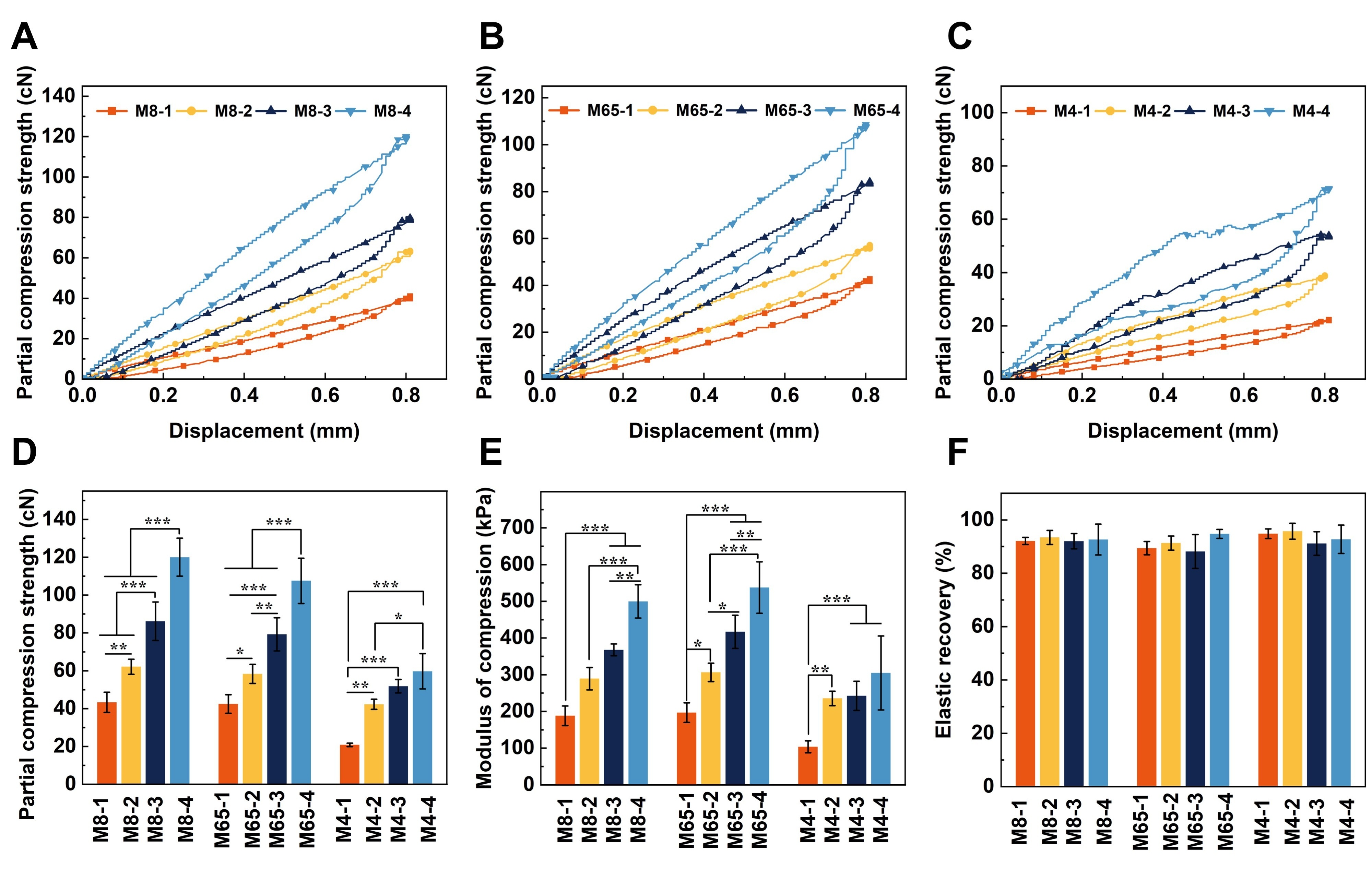


**Figure S5.** Partial compression property of mono-layer stents (n = 5). (A-C) Compression-release curves of group M8, M65 and M4. (D-F) Compression strength, modulus and elastic recovery of mono-layer stents.


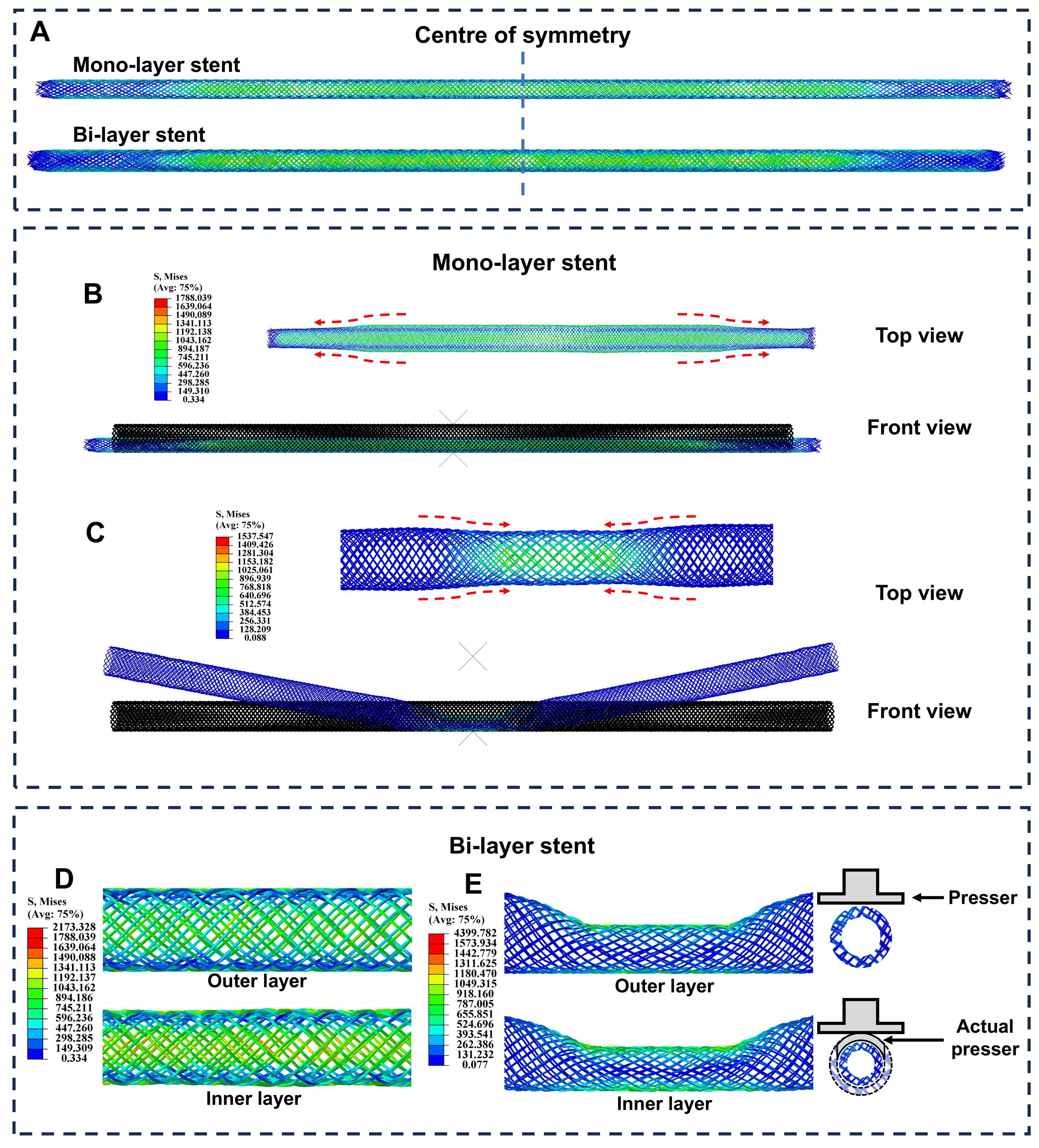


**Figure S6.** Finite element simulation of the mono-layer stent M4-4 and the bi-layer stent B4. (A) Both stent M4-4 and stent B4 displayed a cyclic PPI repetitive layout of low-high-low manner on both sides of the symmetry center. (B) Under plate compression, mono-layer stent M4-4 was flattened and elongated under plate compression. (C) Under partial compression, the diameters of mono-layer stent M4-4 was significantly reduced, while wire slippage, collapse and PPI decrease occurred around the compressed area, which ultimately resulted in the lower radial support. (D, E) The stress distribution of the inner and outer layers of stent B4 under plate and partial compression in finite element simulation.

**Finite element simulation parameters**

Geometry and Assembly: The NiTi braided stents (40 mm) were constructed in Solidworks based on actual measured dimensions and imported into Abaqus as a 3D deformable solid. In Abaqus, we created a rigid presser foot (80 mm × 10 mm) for plate compression and a cylindrical rigid presser foot (diameter 3 mm) for partial compression, and assembled the two presser foots with the underlying rigid plate (80 mm × 10 mm) respectively to form a plate/partial compression finite element analysis model. Material parameters of stent, plate and presser foot were shown in **Table S**7.

The analysis employed explicit dynamic analysis steps.

Element and mesh: The support used C3D8I elements (size: 0.1), whilst the presser foot and underlying rigid plate used C3D10M elements (size: 1).

Interaction: General contact, hard contact, friction coefficient of 0.25.

Reference points RP-1 and RP-2 were defined at the center of mass of the presser foot and underlying rigid plate respectively, and rigid-body constraints were applied to ensure that the presser foot and the underlying rigid plate acted as rigid bodies.

Boundary condition configuration: The underlying rigid plate was fully fixed (U1 = U2 = U3 = UR1 = UR2 = UR3 = 0), and displacement was applied to the presser foot (U1 = U3 = UR1 = UR2 = UR3 = 0; U2 = -0.8 mm).

**Finite element simulation results of the mono-layer and the bi-layer stent**

The variation of stent M4-4 during compression was analyzed by finite element simulation. When subjected to plate compression (**Figure S6**B), the diameter of both ends of stent M4-4 decreased, and the middle section was flattened accompanied by an expansion. The pressure exerted on the stent was transferred to the wire, in which case the component forces in the axial direction drove the wire to slide toward the both ends. At the same time, the wire at the ends of the stent only slid toward the middle section and resisted the slippage of the middle section toward both ends, thereby inhibiting the tendency of the α reduction in certain sections. This resulted in variations of PPI with the midpoint of the stent as the center of symmetry (**Figure S6**A). Under partial compression (**Figure 3**C), stent diameter was significantly reduced, while wire slippage, collapse and PPI decrease occurred around the compressed area, which ultimately resulted in the lower radial support. The simulation results were consistent with the test results.

Following the design and the testing of the bi-layer stent, the variation of stent B4 during compression was analyzed by finite element simulation. The outer stent was compressed first and came into close contact with the inner stent. Similar to stent M4-4, the stent was flattened and elongated under plate compression (**Figure S6**D). The stress was almost applied horizontally to the top/bottom surface (in contact with the presser foot and the base), meanwhile applied vertically to the front/back surface of the stent. (resisting stress from top/bottom surface). The inner layer stent contained more stress bearing segments of the wire in the front/back surface. Moreover, the intensity of stresses experienced by these segments were higher. This result suggested that the inner layer with the lower diameter played a more significant role in stress bearing within the bi-layer stent, and the compression strength decreased as stent diameter increased. Under partial compression, stent B4 demonstrated behavior akin to that of the mono-layer stent M4-4 (**Figure S6**E). Stent B4 achieved a greater partial compression strength than that of stent M4-4 for 473.78%, which could not be attributed individually to the increase in load-bearing structure. Unlike plate compression, the stent was not flattened by partial compression and remained close to its circular shape. The stress applied to the outer layer stent was distributed only in small areas in the top/bottom surface of the stent, while the inner layer stent showed stress distribution expanding towards the front/back surface. For the inner layer, the outer layer actually functioned as the actual presser, which could evenly distribute the stress and transfer it to the top/bottom surface and surrounding areas of the inner layer. For the outer layer, the presence of the inner layer increased the modulus of compression. Moreover, it prevented the wire from sudden slippage and collapse under the pressure.


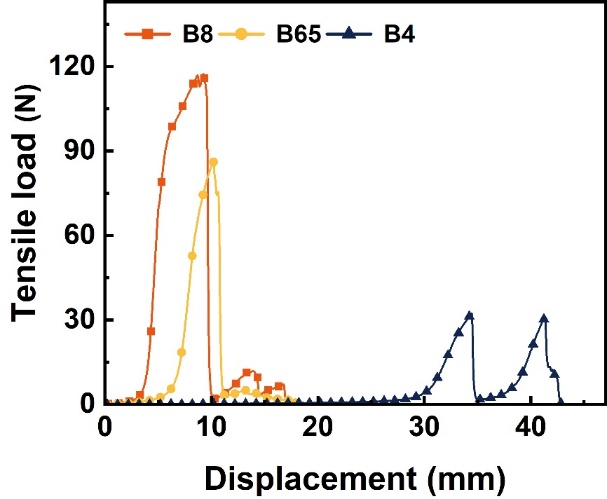


**Figure S7.** Axial tensile curves of bi-layer stents.


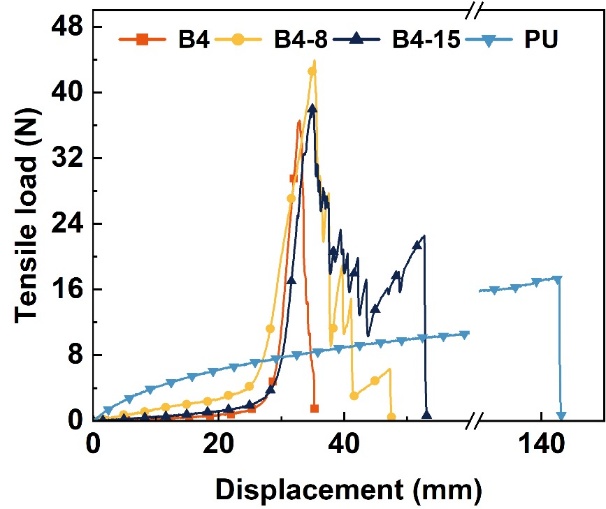


**Figure S8.** Axial tensile curves of interlocked bi-layer stents.


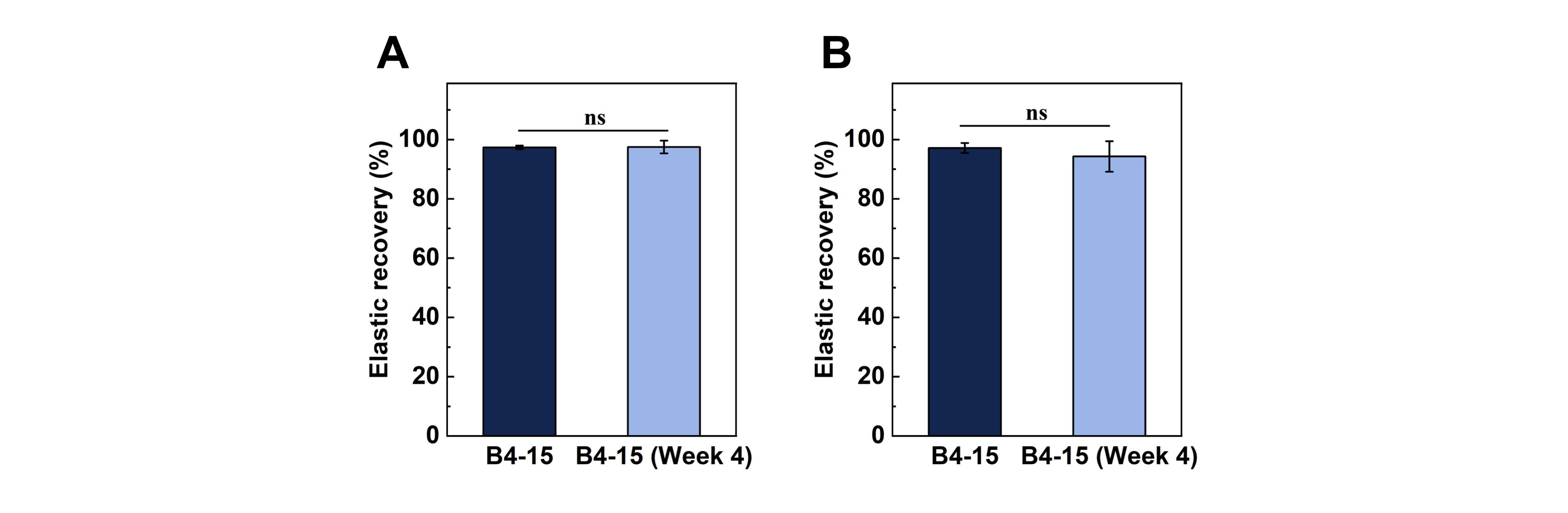


**Figure S9.** Elastic recovery of stent B4-15 and stent B4-15 (Week 4). (A) Under plate compression. (B) Under partial compression.

**Table S1****.** Material composition of NiTi wires as stated in the product inspection report (Ushare Medical Inc.).

| **Composition** | **Ni** | **C** | **O** | **N** | **H** | **Co** | **Cr** | **Cu** | **Fe** | **Nb** | **Ti** |
| --- | --- | --- | --- | --- | --- | --- | --- | --- | --- | --- | --- |
| **Composition SPC (wt%)** | 54.5~57.0 | ≤0.040 | ≤0.040 | ≤0.005 | ≤0.005 | ≤0.050 | ≤0.010 | ≤0.010 | ≤0.050 | ≤0.025 | Balance |
| **Test Result** | 55.58 | 0.015 | 0.032 | 0.004 | 0.00028 | 0.010 | <0.010 | <0.010 | <0.010 | <0.010 | / |

**Table** **S2.** Formation of ureteral stents with three kinds of NiTi wire at different PPI (picks per inch). **S**: Sparse wire arrangement and loose braiding structure with long convergence zone. **M**: Moderate PPI. **O**: Overstocked wire arrangement resulting in strained and even deformed braiding structure.

| **PPI** | **Wire Diameter (mm)** | | |
| --- | --- | --- | --- |
|  | **0.08** | **0.065** | **0.04** |
| 15 | S | S | S |
| 20 | S | S | S |
| 25 | S | S | S |
| 30 | M | S | S |
| 35 | M | S | S |
| 40 | M | M | S |
| 45 | M | M | S |
| 50 | O | M | S |
| 55 | O | M | S |
| 60 | O | O | S |
| 65 | O | O | S |
| 70 | O | O | M |
| 75 | O | O | M |
| 80 | O | O | M |
| 85 | O | O | M |
| 90 | O | O | M |
| 95 | O | O | M |
| 100 | O | O | M |
| 105 | O | O | M |
| 110 | O | O | M |
| 115 | O | O | O |

**Table S3.** Practical structural parameters of 6 Fr mono-layer ureteral stents (n = 5). OD: outer diameter; α: braiding angle, i.e., the angle between the wire and the axial direction of stent.

|  | **M8-1** | **M8-2** | **M8-3** | **M8-4** | **M65-1** | **M65-2** | **M65-3** | **M65-4** | **M4-1** | **M4-2** | **M4-3** | **M4-4** |
| --- | --- | --- | --- | --- | --- | --- | --- | --- | --- | --- | --- | --- |
| **OD (mm)** | 1.88 ± 0.02 | 1.89 ± 0.03 | 1.92 ± 0.04 | 1.98 ± 0.04 | 1.83 ± 0.01 | 1.82 ± 0.02 | 1.83 ± 0.03 | 1.89 ± 0.05 | 1.80 ± 0.02 | 1.79 ± 0.01 | 1.76 ± 0.01 | 1.74 ± 0.01 |
| **Wall (mm)** | 0.14 ± 0.01 | 0.15 ± 0.01 | 0.16 ± 0.02 | 0.19 ± 0.02 | 0.12 ± 0.01 | 0.11 ± 0.01 | 0.12 ± 0.01 | 0.14 ± 0.03 | 0.10 ± 0.01 | 0.09 ± 0.01 | 0.08 ± 0.01 | 0.07 ± 0.01 |
| **2α (**°**)** | 44.40 ± 4.36 | 48.42 ± 4.27 | 57.67 ± 5.98 | 66.99 ± 5.75 | 52.33 ± 4.48 | 58.30 ± 4.80 | 62.66 ± 3.81 | 79.54 ± 6.94 | 72.38 ± 3.43 | 92.23 ± 2.45 | 104.34 ± 1.75 | 107.41 ± 2.27 |
| **Pitch (mm)** | 14.61 ± 0.22 | 12.81 ± 0.08 | 11.31 ± 0.32 | 10.00 ± 0.18 | 11.23 ± 0.05 | 10.21 ± 0.16 | 8.79 ± 0.35 | 8.41 ± 0.15 | 7.10 ± 0.15 | 4.97 ± 0.08 | 4.15 ± 0.04 | 3.84 ± 0.01 |
| **PPI (/inch)** | 28.50 ± 0.71 | 31.60 ± 0.55 | 35.70 ± 0.45 | 39.30 ± 0.45 | 35.90 ± 0.74 | 39.80 ± 0.84 | 45.40 ± 0.55 | 47.60 ± 0.55 | 57.10 ± 0.96 | 81.40 ± 1.14 | 96.20 ± 1.10 | 104.88 ± 0.38 |

**Table S4.** Practical structural parameters of bi-layer ureteral stents (n = 5).

|  | **B8** | **B65** | **B4** |
| --- | --- | --- | --- |
| **OD (mm)** | 2.07 ± 0.02 | 2.03 ± 0.05 | 1.87 ± 0.03 |
| **Wall (mm)** | 0.47 ± 0.02 | 0.43 ± 0.05 | 0.27 ± 0.03 |
| **2α (°)** | 128.18 ± 2.91 | 127.31 ± 2.39 | 113.85 ± 4.76 |
| **Pitch (mm)** | 3.34 ± 0.18 | 3.08 ± 0.06 | 3.69 ± 0.19 |
| **PPI (/inch)** | 119.50 ± 1.73 | 126.20 ± 3.96 | 105.20 ± 11.78 |

**Table S5.** Practical structural parameters of interlocked bi-layer ureteral stents compared to stent B4 (n = 5).

|  | **B4** | **B4-8** | **B4-15** |
| --- | --- | --- | --- |
| **OD (mm)** | 1.87 ± 0.03 | 1.85 ± 0.03 | 1.91 ± 0.03 |
| **Wall (mm)** | 0.27 ± 0.03 | 0.25 ± 0.03 | 0.31 ± 0.03 |
| **Pitch (mm)** | 3.69 ± 0.19 | 3.42 ± 0.06 | 3.47 ± 0.09 |
| **PPI (/inch)** | 105.20 ± 11.78 | 108.20 ± 1.30 | 104.80 ± 2.80 |

**Table S6** Drainage properties of three stents under compression of constant distances (n = 3).

| **Calibre** | **Initial flow rate**  **(mL/min)** | **Sample** | **0 mm**  **Compression** | **0.8 mm** **Compression** | **1.0mm** **Compression** |
| --- | --- | --- | --- | --- | --- |
| **6F** | 11.08 ± 0.54 | B4 | 10.64 ± 0.19 | 5.84 ± 0.20 | 2.78 ± 0.12 |
| **6F** | 11.08 ± 0.54 | B4-15 | 10.60 ± 0.15 | 5.93 ± 0.18 | 2.73 ± 0.08 |
| **6F** | 11.08 ± 0.54 | PU | 4.44 ± 0.18 | 1.33 ± 0.08 | 0.12 ± 0.01 |
| **6F** | 2.98 ± 0.04 | B4 | 3.01 ± 0.15 | 2.37 ± 0.10 | 1.07 ± 0.05 |
| **6F** | 2.98 ± 0.04 | B4-15 | 2.98 ± 0.07 | 2.43 ± 0.12 | 1.05 ± 0.07 |
| **6F** | 2.98 ± 0.04 | PU | 1.12 ± 0.07 | 0.32 ± 0.02 | 0.00 ± 0.00 |

**Table S7** Material parameters of stent, plate and presser foot

| **Parameters** | **Stent** | **Presser foot/ Plate** |
| --- | --- | --- |
| Density (g/cm^3^) | 6.45 | 7.8 |
| Young's Modulus (MPa) | - | 210000 |
| Poisson's Ratio | 0.3 | 0.25 |
| Austenite Young's Modulus (MPa) | 43000 | - |
| Martensite Young's Modulus (MPa) | 29000 | - |
| Transformation Strain | 0.0442 | - |
| Loading Stress: Start of Austenite to Martensite (MPa) | 540 | - |
| Loading Stress: End of Austenite to Martensite (MPa) | 540 | - |
| Unloading Stress: Start of Martensite to Austenite (MPa) | 100 | - |
| Unloading Stress: End of Martensite to Austenite (MPa) | 100 | - |

1. * Lu Wang: wanglu@dhu.edu.cn

   * Fan Zhao: zhaofan@dhu.edu.cn

   1 Key Laboratory of Textile Science & Technology of Ministry of Education, College of Textiles, Donghua University, Shanghai 201620, China

   2 Key Laboratory of Textile Industry for Biomedical Textile Materials and Technology, Donghua University, Shanghai 201620, China

   3 Shanghai Frontiers Science Center of Advanced Textiles, College of Textiles, Donghua University, Shanghai 201620, China

   4 Honest Medical China Co., Ltd., Guangdong 519000, China

   5 Ushare Medical Inc., Guangdong 519040, China [↑](#footnote-ref-1)
